# ﻿New species of the Brachyglutine genus *Panabachia* Park (Coleoptera, Staphylinidae, Pselaphinae) from Ecuador

**DOI:** 10.3897/zookeys.1254.158319

**Published:** 2025-10-02

**Authors:** Sofia I. Muñoz-Tobar, Michael S. Caterino

**Affiliations:** 1 Instituto Nacional de Biodiversidad, Quito, Ecuador Instituto Nacional de Biodiversidad Quito Ecuador; 2 Department of Plant & Environmental Sciences, Clemson University, Clemson, SC 29634, USA Clemson University Clemson United States of America

**Keywords:** Ecuador, montane forest, new species, *
Panabachia
*, páramo, Staphylinidae

## Abstract

Twenty-two new species of *Panabachia* Park (Coleoptera: Staphylinidae: Pselaphinae: Brachyglutini: Brachyglutina), are described from montane forest and páramo habitats of Ecuador: *Panabachia pahuma***sp. nov.**, *P. trifecta***sp. nov.**, *P. inornata***sp. nov.**, *P. amica***sp. nov.**, *P. winku***sp. nov.**, *P. ayauma***sp. nov.**, *P. pastazae***sp. nov.**, *P. romeroi***sp. nov.**, *P. uktu***sp. nov.**, *P. salebrosa***sp. nov.**, *P. urbana***sp. nov.**, *P. carltoni***sp. nov.**, *P. falini***sp. nov.**, *P. papallacta***sp. nov.**, *P. ananay***sp. nov.**, *P. cayambi***sp. nov.**, *P. cryptica***sp. nov.**, *P. caranqui***sp. nov.**, *P. patera***sp. nov.**, *P. vigilans***sp. nov.**, *P. perdita***sp. nov.**, and *P. ambulans***sp. nov.** Only two species of the genus have been previously described, from Panamanian and Guatemalan lowlands. But, based on unpublished records, the genus is now known to occur from Mexico to Bolivia, and includes several additional undescribed species. These new species exhibit important variability in the diversity in male secondary sexual characters. Images of the habitus, key characters and aedeagus are provided for the new species.

## Abstract (es)

Veintidós nuevas especies de *Panabachia* Park (Coleoptera: Staphylinidae: Pselaphinae: Brachyglutini: Brachyglutina), de bosque montano y páramo Ecuatoriano, son descritas: *Panabachia pahuma***sp. nov**., *P. trifecta***sp. nov.**, *P. inornata***sp. nov.**, *P. amica***sp. nov.**, *P. winku***sp. nov.**, *P. ayauma***sp. nov.**, *P. pastazae***sp. nov.**, *P. romeroi***sp. nov.**, *P. uktu***sp. nov.**, *P. salebrosa***sp. nov.**, *P. urbana***sp. nov.**, *P. carltoni***sp. nov.**, *P. falini***sp. nov.**, *P. papallacta* sp. nov., *P. ananay***sp. nov.**, *P. cayambi***sp. nov.**, *P. cryptica***sp. nov.**, *P. caranqui* sp. nov., *P. patera***sp. nov.**, *P. vigilans***sp. nov.**, *P. perdita***sp. nov.** y *P. ambulans***sp. nov.** Previamente, solo se habían descrito dos especies, de tierras bajas de Panamá y Guatemala. Sin embargo, el género se registra desde México hasta Bolivia, con varias especies sin describir. Estas nuevas especies presentan importante variabilidad en los caracteres secundarios del macho. En este estudio se proporcionan fotografías del hábito, caracteres claves y del edeago de las nuevas especies.

## ﻿Introduction

The neotropical fauna of the rove beetle subfamily Pselaphinae is an incredibly diverse one. With more than 1600 known species (Asenjo et al. 2019) it ranks third in neotropical species richness among subfamilies of Staphylinidae, a family that represents one of, if not the largest family of animals on Earth. Yet these described species represent only the tip of an unfathomably large iceberg, with numerous hyperdiverse countries almost completely unknown. Ecuador, for example, hosts only 16 named species of Pselaphinae (unpublished data), while the much smaller but better-surveyed country Panama is known to host more than 500 (Chandler 1992). Neighboring Colombia is known to have at least 88 species of Pselaphinae (Newton et al. 2005). But surveys of even a single site in lowland Ecuador turned up 178 Pselaphine species, while estimating at least a third more (Carlton et al. 2004). At present, all that can be confidently said about pselaphine diversity in any particular neotropical area is that it is undoubtedly much greater than known.

In this paper we explore the diversity in a single pselaphine genus, *Panabachia*, in a single country, Ecuador. *Panabachia* was named by Park (1942) for the Panamanian species *Bryaxis vulnerata* Sharp, based on the unique excavations of the pronotum, distinguishing it from the (purportedly) related genus *Reichenbachia.* Park did not immediately note that Sharp had suggested a close relationship with his *Bryaxis impressicollis*, described in the same paper with similar pronotal modifications. But he later moved this species, described from Guatemala, into *Panabachia* as well (Park 1945). Since that time no additional species have been named or moved to *Panabachia*, and the genus has only been mentioned in catalogs and checklists. Chandler (in Navarrete-Heredia et al. 2002) gave the distribution of the genus, based on still-undescribed species, as Mexico to Bolivia. Otherwise, little new data has been presented on the genus in many decades.

In 2020, as part of a larger study of beetle diversity in high elevation páramo habitats in Ecuador (e.g. Muñoz-Tobar 2019; Muñoz-Tobar and Caterino 2019), Muñoz-Tobar and Caterino (2020) explored the potential species diversity in numerous newly discovered populations of *Panabachia* beetles. Multilocus sequencing and preliminary morphological study suggested as many as 17 different species, revealing a high-elevation radiation in this poorly known group. Further recent collecting at other Páramo sites as well as some in higher montane forest have revealed additional species. Although it is evident that the species we have found represent only the beginning of a substantial and diverse fauna, it will be valuable to begin to document some of the morphological breadth, as well as call attention to this largely high elevation-restricted diversification. Therefore, below we describe 22 new species of *Panabachia* for which species status is well-supported and for which males are available.

## ﻿Materials and methods

### ﻿Field sites and sampling

**Figure 1.**
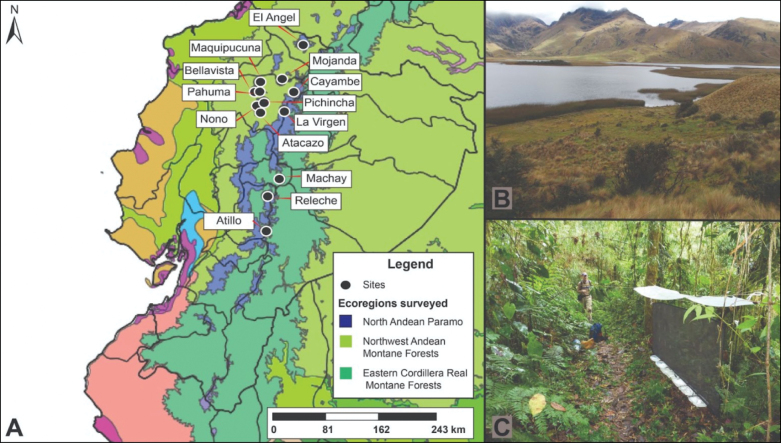
A. Map of the sites where *Panabachia* were collected in the Ecuadorian Andes. Examples of habitat of *Panabachia* species: B. Grassland and shrub páramo in Atillo, province of Chimborazo and C Montane forest in El Pahuma Orchid Reserve, province of Pichincha.

Specimens used in this study were obtained mainly from leaf litter samples, from 13 sites across cloud forest and páramo of Ecuador (Fig. 1). The sampling of the sites was part of three research projects focused on describing beetle diversity in Ecuador. Collecting permits are the following: 0020-FAU-MA-DPO-PNY, MAE-DNG-ARGG-CM-2014-004, and MAATE-DBI-CM--2022-0255. Additional samples were obtained from deposited material at the Instituto Nacional de Biodiversidad/Museo Ecuatoriano de Ciencias Naturales (**MECN-EN**), Museo de Zoología de la Pontificia Universidad Católica del Ecuador Invertebrados (**QCAZ-I**), Museo de Zoología de la Universidad San Francisco de Quito (**ZSFQ**), and Louisiana State Arthropod Museum (**LSAM**).

Morphology was studied using an Olympus stereo-microscope (Olympus SZ61R), genitalia was examined using temporary slide mounts in glycerin, with the use of compound microcopy, as well as photographic images taken with a Macropod Pro 3D (Macroscopic Solutions, CT, USA). The majority of specimens were cleared during DNA extractions, with the use of tissue digestion buffers and proteinase K. The aedeagus was extracted through the abdominal apex, after enzymatic digestion. All extracted specimens were mounted, labelled with extraction numbers, and deposited in public repositories as detailed under ‘type material’ sections below.

### ﻿Measurements

Measurements were taken using photographic material generated with a digital camera (The Imaging Source DFK23UX236) adapted to the stereoscope and analyzed in ImageJ (Schneider *et al*. 2012). At least three individuals per species, females and males, were measured when available (see Table 1). Head length (**HL**) was measured from the clypeal margin to the upper anterior edge of the neck constriction (ignoring the neck); pronotal length (**PnL**) was measured along the midline; pronotal width (**PnW**) was the maximum width, near the midline; elytral length (**EL**) was measured along the suture from the base of the scutellum to the apex of the suture; elytral width (**EW**) was the maximum width, total abdomen length (**AL**) was measured laterally in a straight line from the base of the 1^st^ ventrite to the apex of the last tergite (ignoring telescopy and/or curvature); total length (**TL**) was calculated as head length + pronotum length + elytral length + abdomen length.

**Table 1.** Average (where n > 1) measurements taken in mm, for important body dimensions.

	n	HL	PnL	PnW	EL	EW	AL	TL
*P. pahuma*	1	0.21	0.31	0.34	0.50	0.33	0.42	1.44
*P. trifecta*	1	0.35	0.35	0.37	0.58	0.47	0.30	1.58
*P. inornata*	3	0.32	0.30	0.38	0.55	0.42	0.45	1.62
*P. amica*	1	0.27	0.33	0.46	0.62	0.49	0.42	1.64
*P. winku*	3	0.22	0.35	0.38	0.58	0.37	0.45	1.59
*P. ayauma*	3	0.24	0.30	0.37	0.53	0.39	0.36	1.38
*P. pastazae*	1	0.24	0.27	0.28	0.40	0.32	0.21	1.12
*P. romeroi*	3	0.24	0.31	0.31	0.48	0.36	0.33	1.36
*P. uktu*	4	0.26	0.35	0.37	0.58	0.41	0.38	1.52
*P. salebrosa*	3	0.27	0.37	0.34	0.62	0.39	0.49	1.63
*P. urbana*	3	0.20	0.34	0.37	0.47	0.39	0.47	1.49
*P. carltoni*	2	0.25	0.30	0.32	0.43	0.33	0.37	1.34
*P. falini*	2	0.24	0.35	0.32	0.47	0.35	0.59	1.64
*P. papallacta*	2	0.28	0.30	0.41	0.57	0.31	0.44	1.59
*P. ananay*	3	0.28	0.31	0.34	0.57	0.38	0.41	1.57
*P. cayambi*	3	0.27	0.28	0.37	0.57	0.39	0.41	1.53
*P. cryptica*	2	0.28	0.29	0.33	0.60	0.42	0.40	1.56
*P. caranqui*	1	0.28	0.30	0.37	0.60	0.39	0.45	1.63
*P. patera*	2	0.26	0.36	0.31	0.48	0.38	0.42	1.52
*P. vigilans*	1	0.23	0.38	0.30	0.43	0.34	0.61	1.65
*P. perdita*	1	0.21	0.30	0.31	0.49	0.38	0.48	1.48
*P. ambulans*	1	0.23	0.40	0.47	0.60	0.38	0.65	1.88

## ﻿Taxonomic account

### ﻿*Checklist of the new species* (informally grouped by distinctive characters):

Short flat median lobe:

1. *P. pahuma* sp. nov.
2. *P. trifecta* sp. nov.
3. *P. inornata* sp. nov.
4. *P. amica* sp. nov.
5. *P. winku* sp. nov.
6. *P. ayauma* sp. nov.
7. *P. pastazae* sp. nov.
8. *P. romeroi* sp. nov.
9. *P. uktu* sp. nov.
10. *P. salebrosa* sp. nov.
11. *P. urbana* sp. nov.
12. *P. carltoni* sp. nov.
13. *P. falini* sp. nov.

Longer, curved median lobe:

1. *P. papallacta* sp. nov.
2. *P. ananay* sp. nov.
3. *P. cayambi* sp. nov.
4. *P. cryptica* sp. nov.
5. *P. caranqui* sp. nov.
6. *P. patera* sp. nov.

Genitalia outliers:

1. *P. vigilans* sp. nov.
2. *P. perdita* sp. nov.
3. *P. ambulans* sp. nov.

### ﻿Key to the species of *Panabachia* (males only)

**Table:** 

1	With pronotal modifications including setose pits, depressions, tubercles, or horns; if pronotum unmodified, then ultimate abdominal ventrites slightly depressed or with setose margins (males)	**2**
–	Pronotum lacking modifications and terminal abdominal ventrites simply convex (females)	**not keyed further**
2	Pronotum lacking secondary modifications	**3**
–	Pronotum with distinct secondary modifications	**7**
3	Pronotum widest, angulate in basal 1/2 (Fig. 7G)	***P. perdita***
–	Pronotum widest in distal 1/2, sides rounded	**4**
4	Male flightless (humeri evenly sloped to base); protarsi modified, widened and scaled (Fig. 9); known only from Atillo (Chimborazo Province)	***P. ambulans***
–	Male winged (humeri roundly subquadrate), known from the northern Ecuadorian Andes (Pichincha and Imbabura)	**5**
5	Dorsum rather densely and uniformly setose; known from Pichincha	***P. urbana***
–	Parts of dorsum, particularly head and pronotum, sparsely setose; known from Imbabura	**6**
6	Male last sternite (Fig. 5O) transverse, convex, sparsely setose, with dense row of 6 flattened setae at middle of apical margin; male apical tergite (Fig. 5N) transverse, slightly depressed in the middle	***P. caranqui***
–	Male last sternite transverse (Fig. 5K, L), setose, with a forked process along the inner basal margin, apical margin sinuous, with denser setae at middle; male apical tergite transverse, apically truncate, setose along posterior margin, slightly emarginate in the middle	***P. inornata***
7	Male pronotum with 1 or closely adjacent pair of median impressions, but lacking secondary anterolateral impressions	**8**
–	Male pronotum with 1 or 2 median impressions in addition to pair of anterolateral discal impressions	**13**
8	Male pronotum with curving median depression and more densely setose regions posterolaterad	**9**
–	Median depression varied in shape, but never with more densely setose regions posterolaterad	**10**
9	Male pronotum with median depression (Fig. 4H) nearly V-shaped, with common median area diverging to anterior corners; pronotum strongly narrowed anterad, nearly with apical collar; densely setose regions at sides larger	***P. romeroi***
–	Male pronotum with median depression (Fig. 4G) transverse, laterally curving only weakly forward; pronotal sides evenly curved anteriorly and posteriorly, without apical collar; densely setose regions at sides of pronotal disk smaller	***P. pastazae***
10	Male pronotum (Fig. 4D) widest at base, median depressions located close to posterior margin	***P. amica***
–	Male pronotum widest near middle, median depressions located in anterior 1/2 of disk	**11**
11	Male pronotal sides finely angulate at middle; median depression (Fig. 4M) with paired combs of long, curved setae extending posterad from anterior edge	***P. falini***
–	Male pronotal sides rounded; median depression with few or single anterior setae, not with separated pair or clusters of setae	**12**
12	Anteromedian edge of pronotal depression elevated as a median horn (best seen in lateral view); median depression (Fig. 4A) almost subdivided along midline into pair of midlateral depressions	***P. pahuma***
–	Anteromedian edge of pronotal depression not elevated into median horn; median depression (Fig. 4E) not subdivided at middle, forming a continuous transverse depression	***P. winku***
13	Genal carinae well developed behind eyes (Fig. 5J, M)	**14**
–	Genae not produced as carinae behind eyes	**15**
14	Male pronotum with a pair of adjacent median depressions (Fig. 7A), divided by a thin carina, with 3 short distinct rows of setae at the middle and outer edges; posterolateral corners of male pronotum swollen, but not angulate	***P. papallacta***
–	Male pronotum with larger central median depression (Fig. 7B), with setal patches at lateral corners and divergent tufts on either side of anterior edge; posterolateral corners of male pronotum strongly angulate	***P. ananay***
15	Secondary, anterolateral depressions of male pronotum delimited by distinct, raised carinae	**16**
–	Secondary depressions of male pronotum simple, not delimited by carinae; entire pronotum clearly wider than long	**17**
16	Secondary depressions of male pronotum (Fig. 4L) small, elongate oval, and located anterolateral to transverse median pronotal depression; pronotum slightly longer than wide, slightly prolonged anteriorly	***P. carltoni***
–	Secondary depressions of male pronotum (Fig. 7F) larger, more nearly circular, and located posterolaterad to more narrowly median pronotal depression	***P. vigilans***
17	Median pronotal depression very large, extending onto basal 1/2 of pronotum such that basal median pronotal fovea is within the depression (Figs 4F, 7E)	**18**
–	Median pronotal depression more limited in extent, such that the basal median fovea is free, on a convex portion of disk	**19**
18	Male pronotum (Fig. 7E) wider at midline, sides more or less evenly rounded, broadly longitudinally depressed, with wide, mustache-like comb of setae along anterior edge of depression	***P. ayauma***
–	Male pronotum (Fig. 4F) narrow, elongate, subrectangular; median depression nearly as wide as pronotum, anterior edge with small pair of diverging setal clusters	***P. patera***
19	Male pronotal depression with distinct clusters of setae near its basal midline	**20**
–	Male pronotal depression with only sparse setae	**21**
20	Secondary lateral depressions of male pronotum (Fig. 7C) small and located directly at sides of main median depression; entire posterior margin of median pronotal depression fringed with setae	***P. cayambi***
–	Secondary lateral depressions of male pronotum (Fig. 7D) located posterolaterad main median depression; posterior margin of median depression with short rows of setae at lateral corners of depression	***P. cryptica***
21	Anterior marginal portion of pronotal disk (Fig. 4B) transversely convex, with distinct posterior margin overlapping median and lateral depressions, this swelling relatively densely punctate and setose	***P. trifecta***
–	Anterior marginal portion of pronotum not modified, lacking distinct posterior marginal ridge and not more densely setose or punctate than rest of pronotal disk	**22**
22	Male pronotal depression (Fig. 4I) a very small oval present just behind anterior margin, with interior circular carina enclosing small setose region	***P* . *uktu***
–	Male pronotal depression (Fig. 4J) comprising 2 weakly separated, adjacent, shallow depressions, lacking any carinae	***P. salebrosa***

### ﻿Species descriptions

#### ﻿ Panabachia

Taxon classificationAnimaliaColeopteraStaphylinidae

Park, 1942

72AB3E63-E369-5B10-AB4F-A73DE3D970E9

##### Type species.

*Bryaxis vulnerata* Sharp, 1887, designated by Park (1942).

##### Diagnosis.

The genus *Panabachia* was established principally to recognize the sexually dimorphic male pronotal modification of the type species (*Bryaxis vulnerata* Sharp, 1887, also once placed in *Reichenbachia* Raffray 1904]). They are otherwise rather generalized Brachyglutini, with a median longitudinal gular carina, 11-segmented antennae, two basal carinae on the first visible abdominal segment (usually close together and diverging), and subcontiguous mesocoxae. Several new species described herein do not exhibit pronotal dimorphism, but are unambiguously associated by other characters.

**Figure 2.**
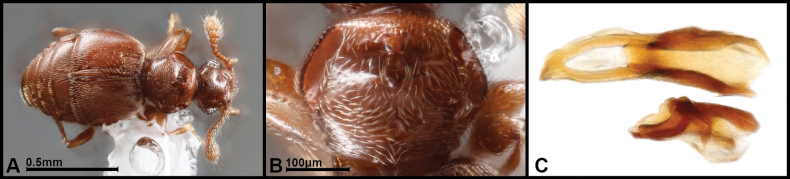
Panabachia near vulnerata (the type of the genus). A. dorsal habitus; B. pronotum; C. aedeagus (one accessory sclerite missing).

No male genitalia have ever been described for the genus. The male genitalia among the new species falls into two very distinct classes. One, represented by only two species, has a short broadly rounded aedeagus bearing a transverse basal bridge. The other typically exhibits an elongate median lobe (incorporating a narrow basal bulb), usually thin parameres, and, separately, a highly specialized pair of articulating accessory sclerites in the distal abdominal membrane. Simple forms of these constitute narrow, flat sclerites, but they become elaborated into complex, coiled, and possibly spring-like structures that probably participate in the extrusion/retraction of the genitalia. We cannot be 100% certain which of these major genitalic forms corresponds to the type (*P. vulnerata*), as its male genitalia has never been illustrated. However, we have dissected a specimen from Costa Rica (LSAM) that appears externally to be very similar to this species (Fig. 2A, B), perhaps conspecific, and its male genitalia represents the more common form, with an elongate median lobe flanked by separate accessory sclerites (Fig. 2C). Among those with distinct accessory sclerites, the male genitalia of the species can be further subdivided into two distinctive forms, one in which the median lobe narrows beyond the basal foramen, and has the apical portion tubular and curving, and one in which the median lobe is shorter and variously flattened beyond the basal foramen. These appear to correspond perfectly to the main clades in Muñoz-Tobar and Caterino (2020), discussed further in the discussion section.

#### ﻿ Panabachia pahuma sp. nov.

Taxon classificationAnimaliaColeopteraStaphylinidae

2F301B66-CA27-5C7E-B3A9-803AA3269A5B

https://zoobank.org/225651C7-F346-4072-9837-F18C2E5B35F4

Figs 3A, 4A, 5A, 6A

##### Type material.

***Holotype*** • ♂ (ZSFQ-i23395): “ECUADOR: Pichincha, 0.0182°N, 78.6372°W, El Pahuma Orchid Res., 28.v-1.vi.2011, FIT, 2200–2400 m. AT1329, M. Caterino, A. Tishechkin” / “Caterino DNA voucher, Ext. MSC-12635, Morphosp. ElP.A.034”; deposited in ZSFQ.

##### Diagnosis.

**Figure 3.**
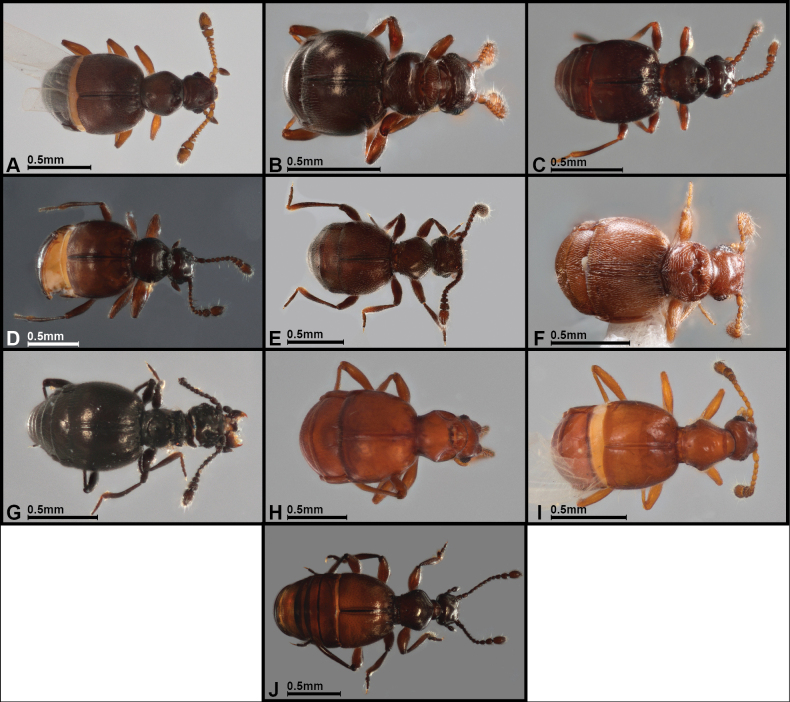
Dorsal habitus of selected *Panabachia* spp. A. *P. pahuma*; B. *P. pastazae*; C. *P. romeroi*; D. *P. salebrosa*; E. *P. urbana*; F. *P. carltoni*; G. *P. patera*; H. *P. vigilans*; I. *P. perdita*; J. *P. ambulans*.

**Figure 4.**
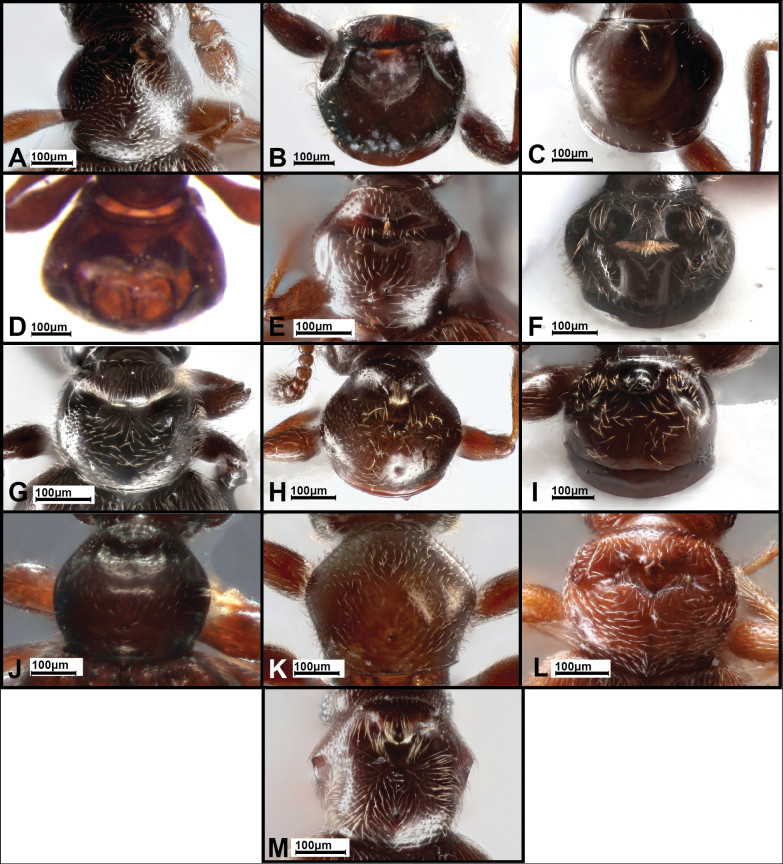
Pronota of *Panabachia* spp. A. *P. pahuma*; B. *P. trifecta*; C. *P. inornata*; D. *P. amica*; E. *P. winku*; F. *P. ayauma*; G. *P. pastazae*; H. *P. romeroi*; I. *P. uktu*; J. *P. salebrosa*; K. *P. urbana*; L. *P. carltoni*; M. *P. falini*.

**Figure 5.**
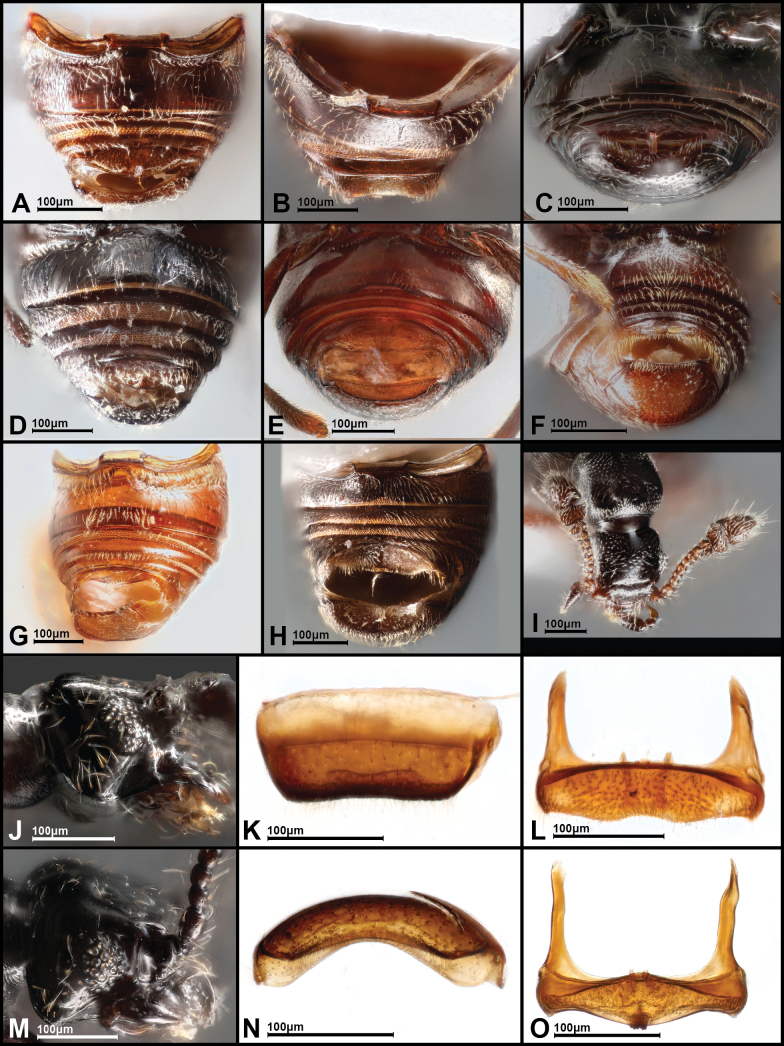
Misc. characters of *Panabachia* spp. A–H. Ventral view of abdomen: A. *P. pahuma*; B. *P. uktu*; C. *P. cayambi*; D. *P. patera*; E. *P. urbana*; F. *P. vigilans*; G. *P. perdita*; H. *P. ambulans*; I. frontal view of head *P. pahuma*; J. Lateral view, head with genal carina of *P. papallacta*; K. Posterior view, tergite 6 of *P. inornata*; L. Ventral view, sternite 6 of *P. inornata*; M. Lateral view, head with genal carina of *P. ananay*; N. Apical view, tergite 6 of *P. caranqui*; O. Ventral view, sternite 6 of *P. caranqui*.

**Figure 6.**
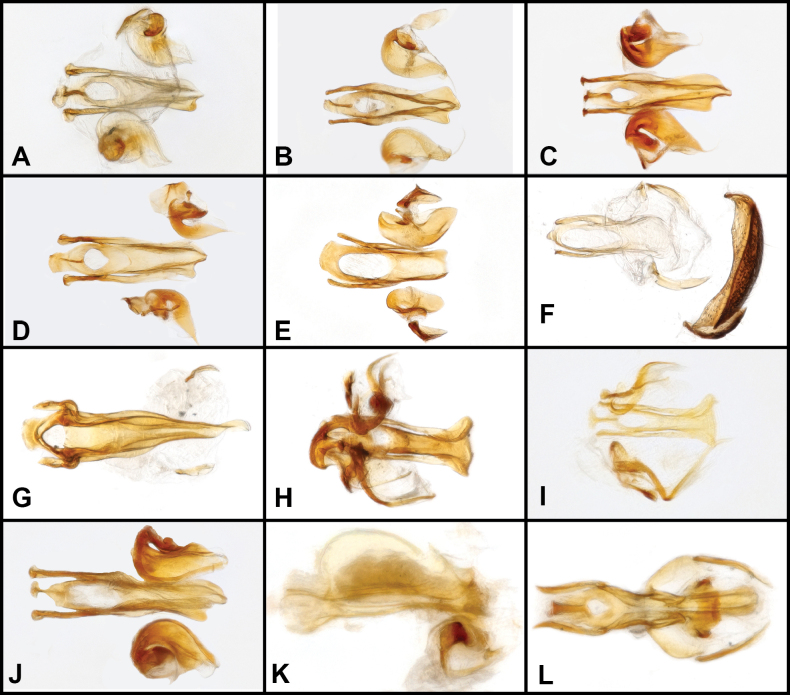
Aedeagus (dorsal view) of *Panabachia* spp. A. *P. pahuma*; B. *P. trifecta*; C. *P. inornata*; D. *P. amica*; E. *P. ayauma*; F. *P. pastazae*; G. *P. romeroi*; H. *P. uktu*; I. *P. salebrosa*; J. *P. urbana*; K. *P. carltoni*; L. *P. falini*.

Head (Figs 3A, 5I) broad, slightly shagreened, with rather thick short, appressed white setae; lateral vertexal foveae well developed, median fovea absent; antennal bases slightly elevated, defined posteriorly by fine bent stria at anterolateral margins; eyes protuberant, round, diameter ~2/3 postocular genal width; antennae rather short, antennomere III slightly longer than wide, antennomeres IV–VI beadlike, rounded, VII–IX increasingly transverse, shorter, antennomere X short, crescent-shaped, entirely excavate on inner margin, antennomere XI ~2 × as long as X, slightly concave on inner basal face, continuous with concavity of X, bluntly rounded apically, with distinct, round setose depression on outer apical surface; male pronotum (Fig. 4A) convex in basal 1/2, lacking lateral basal foveae, with distinct but non-setose median basal fovea; anterior 1/2 of pronotum with bilateral depressions, subcontiguous at middle, anterior edge of depression raised into posteriorly directed, anteriorly triangular, acute process; each elytron with three evenly spaced small basal foveae; sutural stria complete, discal stria absent; legs simple; male last sternite (Fig. 5A) broadly and ovally depressed, rugosely textured, with five thick basally arched setae at middle of apical margin; male apical tergite short, transverse, with broad, densely setose band along posterior margin. Aedeagus (Fig. 6A) elongate, with separate accessory sclerites; parameres separate, knobbed at base, converging and fused with median lobe at middle; median lobe narrow, with small, oval basal foramen, distal portion prolonged into a dorsoventrally flattened blade, margins sinuate to uneven, asymmetrical, subtruncate apical margin; each accessory sclerite with thick, round, sclerotized basal knob, with a thin, semi-spiraled process expanded into triangular apical blade. TL 1.44 mm, EW 0.5 mm.

##### Distribution.

This species is known only from El Pahuma Orchid reserve, Pichincha, Ecuador.

##### Etymology.

This species is named for its type locality.

##### Remarks.

The first four species treated here are very similar in genitalic form, having a flattened aedeagus and free accessory sclerites (Fig. 6A-D). The parameres in these species are all free at the base, fusing with the tegmen around its midpoint (which is also the case in a few species that follow these). The tegmen has a rather small basal foramen, remains wide to near the apex, and has one side entire, the other emarginate. In all of them the accessory sclerites are quite large, with curved inner edges and variously emarginated outer ones.

Given this similarity, the four show remarkably different male pronotal modifications, with one species (*P. inornata*) exhibiting none whatsoever, and their identification based on external characters is straightforward. *Panabachia pahuma* has a unique median ‘horn’ at the anterior margin of the pronotum (Fig. 4A). It is also unusual in occurring lower than most of the others treated in this paper, known from cloud forest habitats (~2500 m) rather than Páramo. There are a few other cloud forest species, but it seems to be a rare excursion from the more typically high elevation habits of this genus.

#### ﻿ Panabachia trifecta sp. nov.

Taxon classificationAnimaliaColeopteraStaphylinidae

2BA4EA83-0F46-5721-92D8-AE2727E061E5

https://zoobank.org/F59C4F44-1DF9-47A0-811B-EC43056E16CE

Figs 4B, 6B

##### Type material.

***Holotype*** • ♂ (QCAZ-I-278811): “ECUADOR: Imbabura, Mojanda, 3715 m, 00°08.710'N, 78°16.753'W, 12-VII-2016, SIMT253, Berlese, S. Muñoz, A. Romero, C. Argos” / “Muñoz DNA Voucher, Ex. SIMT253, Morphosp. Mojanda9”; deposited in QCAZI.

##### Diagnosis.

Head broad, subquadrate, with two vertexal fovea, deep, not setose, closer to eyes on each side than to each other; eyes protuberant but not large, diameter ~1/2 post-ocular genal length; antennal bases slightly elevated, set off by striae; antennae short, antennomere III slightly longer than wide, antennomeres IV–VI weakly transverse, VII–IX increasingly transverse, shorter, antennomeres IX–XI forming loose club, IX and X slightly shorter along inner margins, X 2 × as long as IX, transverse, XI ~3 × as long as X; male pronotum (Fig. 4B) approx. as long as wide, sides almost evenly rounded, widest at middle, evenly depressed along basal margin; anteromedian portion of disk with large cordate depression, with additional, large anterolateral depressions in each corner; anterior marginal portion of pronotal disk transversely convex, with distinct posterior margin overlapping median and lateral depressions, this swelling relatively densely punctate and setose, with row of particularly elongate setae at lateral corners, two anteroprosternal foveae; each elytron with four basal foveae, three foveae evenly spaced and one more distant; legs simple; last abdominal ventrites missing (lost during extraction). Aedeagus (Fig. 6B) elongate, with separate accessory sclerites; parameres separate, thin at base, converging and fused with median lobe basad of middle; median lobe with small, ovate basal foramen, narrowed toward apex, distal portion dorsoventrally flattened, asymmetrical, apical margin obliquely subtruncate on one side, emarginate on the other; each accessory sclerite with thick, sclerotized basal arch, with a thin, distal portion forming a laminate, elongate semicircle with distal style. TL 1.58 mm, EW 0.47 mm.

##### Distribution.

This species is only known from grassland and shrub páramo from Mt. Mojanda, province of Imbabura, Ecuador.

##### Etymology.

The species is named for its tripartite pronotal depressions.

##### Remarks.

As discussed above, this species is similar in male genitalia (and presumably closely related) to the preceding and two following. However, it is readily identified by external characters, with the three anterior pronotal depressions (Fig. 4B) and the unique punctate/setose anterior marginal band in front of them.

#### ﻿ Panabachia inornata sp. nov.

Taxon classificationAnimaliaColeopteraStaphylinidae

E518A5BC-2536-5A93-9363-5DDC33E3A01E

https://zoobank.org/4B126407-90A1-44E3-B794-D741CFC91A4A

Figs 4C, 5K, L, 6C

##### Type material.

***Holotype*** • ♂ (QCAZ-I-278812): “ECUADOR: Imbabura, Mojanda, 3715 m, 00°08.710'N, 78°16.753'W, 28-XII-2016, SIMT297, Berlese, S. Muñoz, A. Romero” / “Muñoz DNA Voucher, Ex. SIMT297, Morphosp. Mojanda11”; deposited in QCAZI. ***Paratypes*** (1♂, 2♀) • 2: same data as holotype • 1: same locality but collected on 12-VII-2016 (SIMT252, SIMT301, SIMT304; QCAZ-I- 278813 to 278815).

##### Diagnosis.

Head broad, subquadrate, with lateral vertexal foveae deep, non-setose, median fovea absent; eyes protuberant, small, diameter ~2/3 of the postocular genal length; two gular fovea present; antennal bases slightly elevated, set off by striae, antennae short, antennomere III slightly longer than wide, antennomere IV–VI beadlike, rounded, VII–IX increasingly transverse, shorter, antennomere IX transverse, 2 × the size of antennomere VIII, antennomere X transverse, apically truncate, 2 × the size of antennomere IX, antennomere XI ~2 × as long as X, setose, rounded, no antennal differences between male and female; male pronotum (Fig. 4C) simple without secondary sexual modifications, slightly wider than long, widest toward front, with very fine median fovea near base; prosternum with two anterior prosternal foveae present; wings present; each elytron with four basal foveae, evenly spaced; male last sternite (Fig. 5K, L) transverse, setose, with a forked process along the inner basal margin, apical margin sinuous, with denser setae at middle; male apical tergite transverse, apically truncate, setose along posterior margin, slightly emarginate in the middle; legs simple. Aedeagus (Fig. 6C) elongate, with separate accessory sclerites; parameres separate, thin at base, converging and fused with median lobe basad of middle; median lobe with small, obovate basal foramen; tegmen rather flat, widest basad middle, constricted, then expanded, apex asymmetrical, apical margin deeply emarginate on one side, mostly outwardly convex on the other, with bluntly produced tip between; each accessory sclerite with thick, sclerotized basal arch, with subrectangular basal part deeply separated from flatter, distal portion forming an elongate blade bearing a distal style. TL 1.53–1.71 mm, EW 0.40–0.43 mm.

##### Distribution.

This species is only known from grassland and shrub páramo from Mt. Mojanda, province of Imbabura, Ecuador.

##### Etymology.

The species is named in recognition of the unmodified male pronotum.

##### Remarks.

Despite a close relationship to the preceding and especially following species on the basis of male genitalic morphology, this species is amply distinguished by its unmodified male pronotum (Fig. 4C).

#### ﻿ Panabachia amica sp. nov.

Taxon classificationAnimaliaColeopteraStaphylinidae

141D5283-8FAF-5A22-A7F3-64675699D0B4

https://zoobank.org/3AEBF430-9896-4962-A89C-1398EEA17500

Figs 4D, 6D

##### Type material.

***Holotype*** • ♂ (QCAZ-I-278816): “ECUADOR: Chimborazo, Páramo de Atillo, 3501 m, 02°11.265'S, 78°31.2601'W, 08-VII-2017, SIMT345, Berlese, S. Muñoz & A. Romero” / “Muñoz DNA Voucher, Ex. SIMT345, Morphosp. Atillo8”; deposited in QCAZI.

##### Diagnosis.

Head broad, with lateral vertexal foveae deep, not setose, closer to eyes on each side than to each other; eyes protuberant but not large, diameter ~2/3 postocular genal length; pair of gular fovea present; antennae short, antennomere III slightly longer than wide, antennomeres IV–VI beadlike, rounded, VII–IX increasingly transverse, shorter, antennomere X ~2 × the size of antennomere IX, transverse, acuminate anteriorly, XI ~3 × as long as X, rounded; male pronotum (Fig. 4D) wider than long, widest at base, evenly narrowed to front; posteromedial portion of disk with deep pair of well-defined, slightly elongate depressions; each posterolateral corner of pronotal disk with shallower depression; prosternum with pair anterior prosternal foveae present; first visible tergite ~2 × length of 2^nd^ or 3^rd^, with close pair of abbreviated longitudinal carinae; each elytron with four basal fovea, three foveae evenly spaced and one distant; legs simple, mesotrochanter slightly toothed in the mid-section; flight wings present. Aedeagus (Fig. 6D) elongate, with separate accessory sclerites; parameres separate, knobbed at base, contacting and articulating with side of median lobe at basal third, thence free distally, with apical setae; median lobe with small, round basal foramen; tegmen rather flat, widest at base and apex, narrower over middle 1/2; apex of tegmen asymmetrical, apical margin deeply emarginate on one side, obliquely rounded on other, with converging strengthening thickenings converting to tip; accessory sclerite almost symmetrical, with thick, sclerotized basal arch deeply separated from flatter, distal portion forming an elongate blade bearing a distal style. TL 1.64 mm, EW 0.49 mm.

##### Distribution.

This species is only known from grassland and shrub páramo around the Atillo lakes, province of Chimborazo, Ecuador.

##### Etymology.

The name of the species refers to the fact that several *Panabachia* species live ‘amicably’ together at this locality.

##### Remarks.

This species follows several with very similar male genitalia, differing markedly, however, in pronotal shape. In this species the pronotum (Fig. 4D) is widest at the base, with large median and smaller lateral basal depressions, very different from most of these that have any pronotal modifications anterior or absent.

#### ﻿ Panabachia winku sp. nov.

Taxon classificationAnimaliaColeopteraStaphylinidae

EBF664E3-3FF0-5BDA-AF84-782E76AB9D10

https://zoobank.org/5E6DEA60-329C-4F6E-91AF-907D4E106199

Fig. 4E

##### Type material.

***Holotype*** • ♂ (QCAZ-I-278844): “ECUADOR: Carchi, El Angel, 18.8 km NW R.B. El Angel, 3300 m, 0°42'24"N, 78°0'25"W, 31 OCT 1999; R. Anderson, ECU1A99 217A, ex: mixed *Polylepis*/shrub litter” / “SM0362673 KUNHM-ENT” / “QCAZ I 278844”; deposited in QCAZI. ***Paratypes*** (3♂, 2♀) • 2: same data as type • 1: same locality as holotype, 3 NOV 1999 • 2: ECUADOR: Carchi, El Angel, 14.1 km NW R.B. El Angel, 3450 m, 0°42'3"N, 77°58'27"W, 2 NOV 1999, mixed *Polylepis* litter (QCAZ-I-278845, 278775, 278776, 278842, 278843).

##### Other material.

2♀: “ECUADOR: Carchi, 0.70587, -77.96642, El Angel, Saladero, 3301m, 26JUL2016, S. Muñoz & R.Tobar, Ex.Berlese, Páramo de frailejones” (QCAZ-I-278817 to 278818) (SIMT294, SIMT295).

##### Diagnosis.

Body dark reddish-brown with faint bronzy sheen, elytra slightly paler than rest of body, very sparsely setose, with most setae recumbent, impunctate; head subquadrate, posterior corners rounded; vertexal foveae deep, not setose, closer to eyes on each side than to each other, with shallow depressions extending anterad; eyes protuberant but not large, diameter ~2/3 postocular genal length; antennal bases slightly raised, with short stria behind, finely emarginate over insertions; pair of gular fovea present; antennae short, antennomere I slightly curved, ~2.5 × as long as wide, II ovoid, ~2/3 length of I, III subconical, slightly longer than wide, IV–VII weakly subquadrate, VIII transverse, shorter, IX–XI forming loose club, XI ~2 × as long as X; male pronotum (Fig. 4E) wider than long, widest just anterad middle, weakly angulate, evenly narrowed to front, base slightly constricted; anteromedial portion of disk with wide, shallowly V-shaped depression, mostly glabrous with small cluster of setae at anterior midpoint; very shallow secondary depressions present on each side of pronotum, posterolaterad main anterior depression; each elytron with four basal fovea, the lateral pair very close together; no subhumeral fovea or stria; flight wings present; prosternum very short, with anterior prosternal foveae at anterior corner of hypomeron; legs simple, mesotrochanter slightly toothed in the mid-section; male metaventrite swollen, with shallow depression and dense cluster of slightly flattened setae between metacoxae; 1^st^ abdominal ventrite with fine median carina; male last abdominal ventrite broadly depressed and setose, opposing margin of last tergite also widened, depressed, and setose. Aedeagus indistinguishable from that of preceding species (Fig. 6D). TL 1.56–1.64 mm, EW 0.35–0.39 mm.

##### Distribution.

This species is only known from páramo habitats of central Carchi province, Ecuador.

##### Etymology.

The species name comes from a Kichwa word meaning ‘curved’, which refers both to the slightly curved pronotal modification and the metatibia.

##### Remarks.

This species has a virtually identical aedeagus to the four preceding species. The pronotum (Fig. 4E), however, is unambiguously distinct, with an almost glabrous, shallowly V-shaped anterior impression with very shallow secondary depressions at the sides. This species likely corresponds to one of two species previously only known from females from the same general area (lineages 10 or 13 from Muñoz-Tobar and Caterino 2020). Morphology suggests that this newly described species may be from lineage 10. Those females are slightly larger, with a more nearly angulate, anteriorly widest pronotum, and show a slight swelling of the metaventrite, more fully expressed in the male. Future work that is able to sequence fresh males of this species will be necessary to evaluate this suggestion.

#### ﻿ Panabachia ayauma sp. nov.

Taxon classificationAnimaliaColeopteraStaphylinidae

B4BE0DCC-F947-5199-A692-97C7B4916280

https://zoobank.org/567B99DC-C051-40A2-A7BB-C49734EBB379

Figs 4F, 6E

##### Type material.

***Holotype*** • ♂ (QCAZ-I-278819): “ECUADOR: Chimborazo, El Releche, 3124 m, 00°38.400'S, 78°30.426'W, 8-VII-2016, SIMT256, Berlese, S. Muñoz & A Romero” / “Muñoz DNA Voucher, Ex. SIMT256, Morphosp. Releche14”; deposited in QCAZI. ***Paratypes*** (4♀) • 2 same data as holotype (SIMT257, SIMT283) (QCAZ-I-278820 to 278821) • 2: same locality as type, but collected on 22-Jul-2017 (SIMT340, SIMT341) (QCAZ-I-278822 to 278823).

##### Other material.

1: same data as type, 22-Jul-2017 (SIMT342), but sample vial included parts of 1 male and 1 female specimen. It is unclear which was sequenced in Muñoz-Tobar and Caterino (2020) (QCAZ-I-278824).

##### Diagnosis.

Head missing (lost during DNA extraction); male pronotum (Fig. 4F) wider than long, widest toward front, sides more or less even rounded, basal margin of disk depressed; most of disk sparsely punctate and with long setae; median portion of disk glabrous, broadly longitudinally depressed from near base to just in front of middle, depression there splits around broad, posteriorly widened and truncate anteromedian ridge that is densely lined with transverse series of diverging setae; anterior depressions on each side of ridge deeply rounded, smooth, with longer setae in irregular series around anterior and lateral edges; laterad these median depressions, each side of pronotum has smaller, round, secondary depression, also with series of setae around lateral margin; elytron with three evenly spaced foveae, no discal stria, apical stria incomplete; male last abdominal ventrite depressed; opposing edge of male last tergite widened, setose; legs simple. Aedeagus (Fig. 6E) broad and flat, with separate accessory sclerites; parameres separate, thin at base, converging and articulated with median lobe basad of middle, free and setose apically; median lobe with broad base, lacking basal apodeme; basal foramen large, ovate; median lobe slightly narrowed toward wide, very shallowly emarginate apex; accessory sclerites moderately asymmetrical, each bipartite, with thinner, flatter median portion and smaller, articulated lateral portion; the median elements weakly spatulate distally, with outer margins knobbed at articulation point; the lateral portions more strongly sclerotized, short, angulate on outer edge, scoop-like on inner apical surface. TL 1.35–1.41 mm, EW 0.37–0.42 mm.

##### Distribution.

This species is only known from subpáramo habitats surrounding the Releche Hacienda, province of Chimborazo, Ecuador.

##### Etymology.

The name of this species comes from the Kichwa, meaning ‘the one that leads the way’.

##### Remarks.

The aedeagus of this species (Fig. 6E), with a broad flattened tegmen, separate parameres, and large accessory sclerites, allies it with all those preceding. The tip of the aedeagus is distinct, being rather simply subtruncate. And the accessory sclerites themselves are subdivided into sclerotized and larger laminate portions. Externally the species is distinguished by a unique male pronotum (Fig. 4F), depressed along much of the midline, opening into deep, paired anterior depressions, with a dense transverse setal series at the split.

#### ﻿ Panabachia pastazae sp. nov.

Taxon classificationAnimaliaColeopteraStaphylinidae

3A50C905-FCDD-590B-A7BE-CA1DC89F755A

https://zoobank.org/D216F449-064E-46CD-839E-5B86AE4B6D7E

Figs 3B, 4G, 6F

##### Type material.

***Holotype*** • ♂ (MCEN-EN 40801): “ECUADOR: Tungurahua, -1.3829, -78.2909, Rio Machay Reserve, 2382 m, 12.XI.2024, M.Caterino, sifted litter” / “Caterino DNA voucher, Ext. MSC-13154, Morphosp. Mch.017”; deposited in MECN.

##### Diagnosis.

Head (Fig. 3B) broad, setose, with vertexal foveae deep, non-setose, closer to eyes on each side than to each other; eyes protuberant, moderately large, diameter approx. equal to postocular genal length; antennal bases elevated slightly elevated, set off by oblique striae; antennae short, antennomere II globose, III subconical, antennomeres IV–VI beadlike, rounded, VII–IX increasingly transverse, IX 2 × the size of antennomere VIII, transverse, antennomere XI ~2 × as long as X, rounded, densely setose, with small depression on inner apex; male pronotum (Fig. 4G) slightly wider than long, sides evenly rounded; disk convex in basal 1/2, with very fine mediobasal fovea, conspicuously clothed with mostly inwardly directed setae, with small, discrete, rugose areas on either side; anterior portion of pronotal disk with wide, arcuate depression, narrowest at middle, slightly broader at sides where depression extends nearly to each anterior corner, depression with denser setae along anterior margin; pronotal disk in front of depression with setae mostly directed posteriorly toward depression; elytra moderately long, convex, with sides rounded, each with just sutural and single lateral dorsobasal fovea, a distinct sutural stria extending posterad from fovea; male last tergite truncate and setose at apex; prosternum short, anterior prosternal foveae present; legs simple. Aedeagus (Fig. 6F) elongate with separate accessory sclerites; parameres free at base, sinuate, converging to tegmen at middle; tegmen flattened, narrowest at base, with large, elongate oval basal foramen, unevenly widened apically, distal third broadly spatulate, apex rounded and weakly sclerotized; accessory sclerites more or less symmetrical, each comprising small sclerotized basal piece articulated with flattened, curved laminate blade with subacute apex. TL 1.12 mm, EW 0.32 mm.

##### Distribution.

This species is only known from montane forest at the Reserve Rio Machay, province of Pastaza, Ecuador.

##### Etymology.

The name of the species comes from the Rio Pastaza watershed where it is so far exclusively found.

##### Remarks.

This species has only been collected in mid-elevation cloud forest, and not páramo habitats. With only a single example known, we cannot say whether this is an exclusive preference, but it sets it apart from most of the other species here. Its rather simple transversely arcuate anterior pronotal sulcus (Fig. 4G) is unique, as is the presence of only two basal elytral foveae. These characters, along with geography, should make it straightforward to identify without resorting to the also unique, spatulate aedeagus (Fig. 6F).

#### ﻿ Panabachia romeroi sp. nov.

Taxon classificationAnimaliaColeopteraStaphylinidae

4583F8E1-3B52-5399-BFCC-1126B684C8D0

https://zoobank.org/0D4FEB19-4FEF-4528-8E36-CDB04224E693

Figs 3C, 4H, 6G

##### Type material.

***Holotype*** • ♂ (QCAZ-I-280223): “ECUADOR: Carchi, 0.70587, -77.96642, El Angel, Saladero, 3301 m, 26JUL2016, S. Muñoz & R. Tobar, Ex. Berlese, Páramo de frailejones” / “Muñoz DNA Voucher, Ex. SIMT289, Morphosp. ElAngel17”; deposited in QCAZI. ***Paratypes*** (1♂, 1♀) • same data as holotype (SIMT289: QCAZ-I-280224 to 280225).

##### Diagnosis.

Head (Fig. 3C) broad, sparsely setose; posterior margin of the head elevated, rounded; two vertexal fovea deep, non-setose, closer to eyes on each side than to each other; eyes protuberant, moderately large, diameter equal to postocular genal length; antennal bases slightly elevated, set off by oblique stria; antennae short, antennomere II globose, III subconical, antennomeres IV–VI beadlike, rounded, VII–IX increasingly transverse, IX 2 × the size of antennomere VIII, transverse, XI ~2 × as long as X, rounded, densely setose, with small depression on inner apex; male pronotum (Fig. 4H) slightly longer than wide, widest just behind middle, strongly narrowed to front, anterior portion of lateral margins slightly concave; disk convex in basal 1/2, with fine mediobasal fovea, mostly smooth, with sparse, fine, setose punctures, slightly denser anteriorly and laterally; anterior portion of disk with widely V-shaped depression, smooth within, the upper arms nearly reaching anterior margin, with long, anteriorly directed setae along posteromedial margin and a denser tuft at the inner base of the V; elytra sparsely setose, each elytron with four evenly spaced foveae, no discal stria, apical stria complete; male last abdominal ventrite entirely depressed, rough, apical margin outwardly subangulate, with recurved setose tooth at apex; opposing surface of male last tergite broadly emarginate, thickened, setose on lower edge; legs simple. Aedeagus (Fig. 6G) broad at base, with wide flat basal rim, flattened, bent abruptly distad ~1/4 from base; parameres free at base, bent inward and fusing with median lobe just distad basal foramen, apices bent in parallel with median lobe, short, free, weakly setose; median lobe narrowing toward apex, with tip upturned, weakly curved, narrowly subacute; accessory sclerites present, free, very reduced, with short, thick basal part and thin, crescent-shaped apical part. TL 1.28–1.41 mm, EW 0.34–0.38 mm.

##### Distribution.

This species is only known from páramo habitats of central Carchi province, Ecuador.

##### Etymology.

We name this species to acknowledge the assistance in the field of Dr. Andrés Romero-Carvajal, who helped collect several specimens described in this manuscript.

#### ﻿ Panabachia uktu sp. nov.

Taxon classificationAnimaliaColeopteraStaphylinidae

F06DF83F-7CAC-5FB9-9D0E-06B0168FF077

https://zoobank.org/C3CFF9D9-BDE4-4382-B519-A3EA1630CFA8

Figs 4I, 5B, 6H

##### Type material.

***Holotype*** • ♂ (QCAZ-I-280226): ECUADOR: Chimborazo, Páramo de Atillo, 3501 m, 02°11.265'S, 78°31.2601'W, 07-VII-2016, Berlese, S. Muñoz & A. Romero” / “Muñoz DNA Voucher, Ex. SIMT344, Morphosp. Atillo15”; deposited in QCAZI. ***Paratypes*** (1♂, 3♀) • same data as holotype (SIMT343, SIMT346, SIMT347, SITM349; QCAZ-I-28022 to 2802230).

##### Diagnosis.

Head broad, sparsely setose, with lateral vertexal foveae deep, not setose, closer to eyes on each side; median fovea absent; eyes protuberant but not large, diameter ~2/3 of postocular genal width; two gular foveae present; antennal bases elevated, set off by oblique stria; antennae short, antennomere III slightly longer than wide, IV–VI beadlike, rounded, VII–IX increasingly transverse, X 2 × the length of antennomere IX, transverse, XI ~2 × as long as X, rounded, densely setose, with depression on inner apex; male pronotum (Fig. 4I) approx. as long as wide, sides just slightly wider in anterior 1/2, weakly constricted basally; disk weakly depressed along basal margin, posterior 2/3 almost evenly convex, with fine mediobasal fovea; most of disk sparsely and finely punctate, with long, slightly flattened setae in punctures; small, oval median depression present just behind anterior margin, with interior circular carina within, setose within this circle and along upper margin of depression; oblique lateral depressions present extending posterolaterad from median depression, these with denser series of setae along anterior and lateral edges; two anteroprosternal foveae present; each elytron with four basal foveae, three foveae evenly spaced and one disjunct, discal stria absent; apical elytral stria incomplete; wings present; male last sternite (Fig. 5B) flattened, setose along apical margin, with small bifid process on apical margin (this is the external manifestation of a larger, internal, forked lever that articulates outward when genitalia are everted), flanked by modified setae; legs simple. Aedeagus (Fig. 6H) with base curved ventrad, flattening out and widened apically, with separate accessory sclerites; parameres long, connected at sides of basal foramen, free to setose apex; median lobe with basal apodeme curved underneath and slightly distad; basal foramen ovate; median lobe slightly narrowed beyond foramen, then expanded widely to subtruncate, brushy apex, with pads beneath; accessory sclerites complex, asymmetrical, associated with base of median lobe rather than apex; left side with short clubbed process at base, and long thin, apically curving distal process; right side with strong basal arch dividing into two thin, curved distal processes, neither as long as thin process of other side. TL 1.34–1.69 mm, EW 0.32–0.47 mm.

##### Distribution.

This species is only known from grassland and shrub páramo around the Atillo lakes, province of Chimborazo, Ecuador.

##### Etymology.

The name of this species comes from a Kichwa word meaning hole, referring to the male pronotal modification.

##### Remarks.

See the following remarks section for details on separating this from the sympatric and closely related *P. salebrosa*.

#### ﻿ Panabachia salebrosa sp. nov.

Taxon classificationAnimaliaColeopteraStaphylinidae

35744E10-A387-5D9F-9A8D-6E402F6E9C0C

https://zoobank.org/FAD844D4-99A7-41D0-BF9A-B5DF82E364E9

Figs 3D, 4J, 6I

##### Type material.

***Holotype*** • ♂ (QCAZ-I-280231): “ECUADOR: Chimborazo, -2.18775, -78.5210, Atillo, 3501 m, 8JUL2017, S. Muñoz & A. Romero, Ex. Berlese” / “Caterino DNA voucher, MSC-13034, Morphosp. Atillo1”; deposited in QCAZI. ***Paratypes*** (3♂) • same general locality as holotype, but collected on 7-Jul-2016 (QCAZ-I-280255-280257).

##### Diagnosis.

Head (Fig. 3D) broad, sparsely setose, with vertexal foveae deep, non-setose, closer to eyes on each side than to each other; median fovea absent; eyes protuberant but not large, diameter ~2/3 of postocular genal width; two gular foveae present; antennal bases elevated, set off by oblique stria; antennae short, antennomere III slightly longer than wide, IV–VI beadlike, rounded, VII–IX increasingly transverse, X 2 × the length of IX, transverse, bluntly acuminate, with setose depression on anterior surface, XI ~2 × as long as X, rounded densely setose with depression on inner apex; male pronotum (Fig. 4J) almost as long as wide, sparsely and finely punctate and setose, setae slightly denser in anterior 1/3, widest behind midline, narrower at apex than base, anterior margin slightly rounded; posterior 1/2 of disk evenly convex, with fine median fovea; bifid depression (broad depression slightly constricted along midline) present in anterior third, anterior edge a little more abrupt than posterior; shallower, slightly oblique lateral impressions present alongside median ones; two anteroprosternal fovea present; each elytron with four basal foveae, evenly spaced; elytra sparsely setose, discal stria absent, apical stria incomplete; wings present, male last sternite flattened, transverse, sparsely setose; male apical tergite transverse, slightly convex in the middle, sparsely setose; legs simple. Aedeagus (Fig. 6I) flat, with separate accessory sclerites; parameres separate, thin at base, briefly fused to median lobe subbasally, then free to apex, expanded; median lobe with basal apodeme distinct, T-shaped; basal foramen small, ovate; median lobe slightly narrowed beyond foramen, then expanded widely to weakly rounded, brushy apex, with setose apices of parameres alongside; accessory sclerites complex, asymmetrical, associated with base of median lobe rather than apex; both with tightly coiled base opening into elongate, sinuate distal processes, one much longer and thinner than other. TL 1.31–1.82 mm, EW 0.37–1.41 mm.

##### Distribution.

This species is only known from grassland and shrub páramo around the Atillo lakes, province of Chimborazo, Ecuador.

##### Etymology.

The name of this species refers to the ‘uneven’ anterior portion of the male pronotal disk.

##### Remarks.

This species is sympatric with *P. uktu*, and the two exhibit similar male genitalia (Fig. 6H vs Fig. 6I). Yet there are clear differences in the more basally simpler median lobe of *P. salebrosa*, in its flatter median lobe, and in its more prolonged left accessory sclerite, as well as in minor differences of male pronotal morphology.

#### ﻿ Panabachia urbana sp. nov.

Taxon classificationAnimaliaColeopteraStaphylinidae

5AACE4D2-F64F-54CF-BC2A-EE365C4CA122

https://zoobank.org/DCE584EF-A69D-4954-ADD5-CD314CCED7AE

Figs 3E, 4K, 5E, 6J

##### Type material.

***Holotype*** • ♂ (MECN-EN 40874): “ECUADOR: Pichincha, Quito, Cerro Atacazo, -0.347, -78.611, m, Arbustivo quemado, 01-dic-2018, J. Obregón” / “PTA-196” / “Caterino DNA voucher, Ext. MSC-13316, Morphosp. Panabachia_Atacazo2” / “MECN-EN 40874”; deposited in MECN. ***Paratypes*** (2♂, same locality as holotype) • 1: 1-Nov-2018 • 1: 1-Mar-2019 (MECN-EN 40875 to 40876).

##### Diagnosis.

Body (Fig. 3E) dark reddish, densely covered with short, semierect setae; head broad, subquadrate, densely setose, with lateral vertexal foveae deep, not setose, closer to eyes on each side than to each other; eyes protuberant but not large, diameter slightly greater than postocular genal width; genal carinae not exaggerated; two gular foveae present; antennal bases slightly elevated, set off by oblique stria; antennae short, antennomere I short, cylindrical, II similar in width, ovoid, III–VI, beadlike, rounded, VII–IX increasingly transverse, shorter, X short, XI ~2.5 × as long as X, narrowly rounded, densely setose, with depression on inner apex; no pronotal dimorphism, pronotum (Fig. 4K) slightly wider than long, widest near middle, slightly constricted before the base; median pronotal fovea fine, deeply impressed; each elytron with four basal foveae, evenly spaced; sutural stria complete, discal stria absent; wings absent; 1^st^ abdominal tergite with close, short pair of curved basal carinae; pair of anteroprosternal foveae present; legs simple; 1^st^ male abdominal ventrite (Fig. 5E) weakly emarginate at middle; last male ventrite wide, flattened, densely setose along most of apical 1/2; last male tergite with apical margin wide, setose, similar to opposing ventrite. Aedeagus (Fig. 6J) relatively flat, with basally free parameres and separate accessory sclerites; parameres fused to median lobe at sides of large, elongate oval basal foramen; median lobe with enlarged basal knob, distally widening slightly to asymmetrically bifid apex, with left side emarginate; accessory sclerites slightly asymmetrical, with tightly coiled basal part, and enlarging, laminate distal part with curved and thickened margins. TL 1.38–1.60 mm, EW 0.35–0.41 mm.

##### Distribution.

This species is known only from páramo habitats at Cerro Atacazo, province of Pichincha, Ecuador.

##### Etymology.

This species is named for its proximity to the Quito metropolitan area.

##### Remarks.

This species is sympatric with *P. patera*, but distinctive in numerous characters. *Panabachia urbana* lacks male pronotal dimorphisms (Fig. 4K) (and males can be easily recognized by modifications of the last abdominal ventrite; Fig. 5E), and is also lighter in coloration, a bronzy brown, and broader in shape.

#### ﻿ Panabachia carltoni sp. nov.

Taxon classificationAnimaliaColeopteraStaphylinidae

4A601A15-B4C0-5905-92D4-67E93F285DD1

https://zoobank.org/43F59321-6771-4690-A0D7-8176741C9285

Figs 3F, 4L, 6K

##### Type material.

***Holotype*** • ♂ (MECN-EN 42138): “Ecuador: Pichincha Pr. 50 km NW Quito, Reserva Maquipucuna, #13, elv. 1350 m, 20 Dec.1991, light, C. Carlton, R. Leschen” / “Caterino DNA voucher, Ext. MSC-13326, Morphosp. PanMaq1”; deposited in MECN. ***Paratypes*** (1♂, 1♀) • same data as holotype (MECN-EN 42139 to 42140).

##### Diagnosis.

Body (Fig. 3F) pale brownish-orange, with conspicuous whitish, subrecumbent setae; impunctate; head subquadrate, posterior corners rounded; vertexal foveae deeply impressed; eyes protuberant; antennal bases with very short stria behind; antennae short, basal two antennomeres similar in length, cylindrical, III subconical, IV–VIII short, subquadrate, IX wider, transverse, barely part of club, X–XI conspicuously larger, setose; pronotum broad, sides almost evenly rounded, slightly wider anterad; anterior part of male pronotal disk (Fig. 4L) with transverse depression, bi-arcuate, anterior midpoint pointing posterad over middle, bearing few diverging setae at apex, depression glabrous within, setose particularly along posterior margins; laterad depression are very slightly raised, elongate oval disks, finely rugose but not setose within; posterior portion of pronotal disk with median fovea but otherwise unmodified; elytra with four approximately evenly spaced basal foveae; prosternum very short, hypomeron with anterior foveae; male metaventrite slightly swollen, otherwise unmodified; male last abdominal ventrite short, shallowly transversely depressed; margin of last male tergite unmodified. Aedeagus (Fig. 6K) flattened, with basally separate parameres, and separate accessory sclerites; tegmen with apex expanded, membranous across front; accessory sclerites asymmetrical, one with short blunt tip, other with elongate, apically hooked tip. TL 1.32–1.35 mm, EW 0.33 mm.

##### Distribution.

This species is only known from the Maquipucuna Reserve, ca 35 km NW of Quito. Although several other species occur very close by (3 species at El Pahuma), none appear to be particularly closely related to this one.

##### Etymology.

We name this species to acknowledge the collaboration and assistance (in this and many other projects) of Dr. Christopher Carlton, who helped collect the types of this species.

##### Remarks.

There is limited material of this species, and the male dissection is not well sclerotized nor well cleared. Therefore several details are obscured. Nonetheless it does have several unique features, including the apical prolongation of one of the accessory sclerites. The dorsal velum (visible in Fig. 6K) of the tegmen is possibly unique, but possibly also a remnant of an internalized tergite not extracted in other species.

#### ﻿ Panabachia falini sp. nov.

Taxon classificationAnimaliaColeopteraStaphylinidae

8B3E5927-D502-5A4E-A4F6-696986E2EA41

https://zoobank.org/F52768B0-BE91-452A-898D-F5D685F45B2A

Figs 4M, 6L

##### Type material.

***Holotype*** • ♂ (QCAZ-I-278840): “ECUADOR: Pichincha, Nono, 15.1 km NW, 2000 m, 0°1'58"S, 78°39'19"W, 24–26 OCT 1999; Z. H. Falin, ECU1F99 022, ex: flight intercept trap” / “SM0351637” / “QCAZ-I-278840”; deposited in QCAZI.

##### Other material.

1♀: “ECUADOR: Pichincha, Bellavista Reserve, Ridge Trail, 12 km S Nanegalito, 2250 m,0°0'54"N, 78°40'56"W, 28 OCT 1999; R. Anderson, ECU1A99 211F, ex. Cloud forest litter” (SM0362958/QCAZ-I-278841).

##### Diagnosis.

Body dark orange, very finely setose, setae recumbent, impunctate; head subquadrate, posterior corners rounded, vertex slightly swollen above deeply impressed vertexal foveae; frons with oblique depressions mediad vertexal foveae; antennal bases weakly elevated in front of fine, oblique striae, striae nearly meeting across middle; eyes large, protuberant, diameter ~2 × post-ocular genal length; antennae short, antennomere I slightly curved, ~2 × as long as wide, II subquadrate, ~2/3 length of I, III subconical, slightly longer than wide, IV–VII weakly subquadrate, VIII transverse, shorter, IX–XI forming loose club, XI ~2 × as long as X; male pronotum (Fig. 4M) wider than long, elevated across entire anterior margin, sides angulate at widest point, at sides of anterior elevation; deep triangular impression present behind middle of anterior elevation, with short, dense, transverse cluster of setae projecting backwards from anterior margin just laterad midline, a few longer setae projecting anterad from posterior edge of impression; depression shallowly continued in anterolateral, glabrous depressions; extreme sides of pronotal disk with irregularly ovoid, very shallow, glabrous depressions, their outer/lower edges forming lateral pronotal margin; each elytron with four basal fovea, the lateral pair in a common depression, barely distinguishable; no subhumeral fovea or stria; flight wings present; prosternum very short, with anterior prosternal foveae at anterior corner of hypomeron; legs simple, mesotrochanter slightly dentate at middle of posterior edge; male metaventrite setose posteriorly between metacoxae; male last abdominal ventrite densely setose along distal margin, depressed on either side of a thin, prominent median denticle; terminal male tergite with apical margin widened, flattened, and densely setose. Aedeagus (Fig. 6L) slender, base slightly bulbous, with small, oval basal foramen; parameres free for basal 1/4, then fused with median lobe just beyond foramen; median lobe slightly constricted beyond paramere fusion, dorsally arched, with long median part a flattened cylinder, a shorter, slightly widening dorsal process over it, just shorter, and lateral ‘wings’ with long, curved, articulated rods recurving toward middle at apices. TL 1.6–1.67 mm, EW 0.33–0.36 mm.

##### Distribution.

This species is known from northeastern montane forest in the province of Pichincha, Ecuador.

##### Etymology.

We name this species to acknowledge many years of collaboration and assistance, on this project and numerous others, from Dr. Zachary Falin, collector of the type of this species.

#### ﻿ Panabachia papallacta sp. nov.

Taxon classificationAnimaliaColeopteraStaphylinidae

F6384622-03E4-5A84-8759-A829D135F55E

https://zoobank.org/FB2E93D3-219C-4A2F-B3B4-B74ECFA1B62B

Figs 5J, 7A, 8A

##### Type material.

***Holotype*** • ♂ (QCAZ-I-278835): “ECUADOR: Pichincha, -0.30795, -78.23255, La Virgen, 3694 m, 28JUN2016, S. Muñoz, & R. Tobar, Ex. Berlese, Bosque Polylepis y pajonal” / “Muñoz DNA Voucher, Ex. SIMT246, Morphosp. LaVirgen1”; deposited in QCAZI. ***Paratypes*** (1♂, 4♀) • 1 same data as holotype (SIMT275) • 4: “ECUADOR: Pichincha, -0.32124, -78.21319, La Virgen, 3942 m, 18JDEC2016, S. Muñoz & R. Tobar, Ex. Berlese, Leaf litter & moss” (SIMT305 to 308) (QCAZ-I-278725 to 278829).

**Figure 7.**
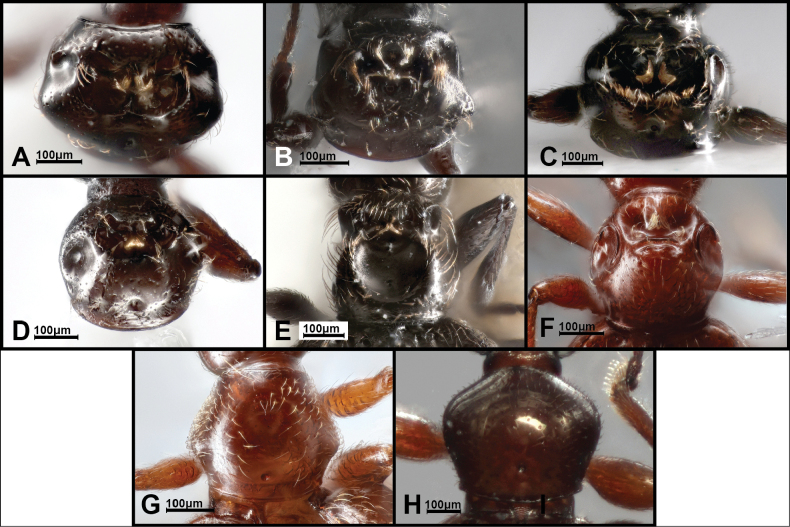
Pronota of *Panabachia* spp. A. *P. papallacta*; B. *P. ananay*; C. *P. cayambi*; D. *P. cryptica*; E. *P. patera*; F. *P. vigilans*; G. *P. perdita*; H. *P. ambulans*.

**Figure 8.**
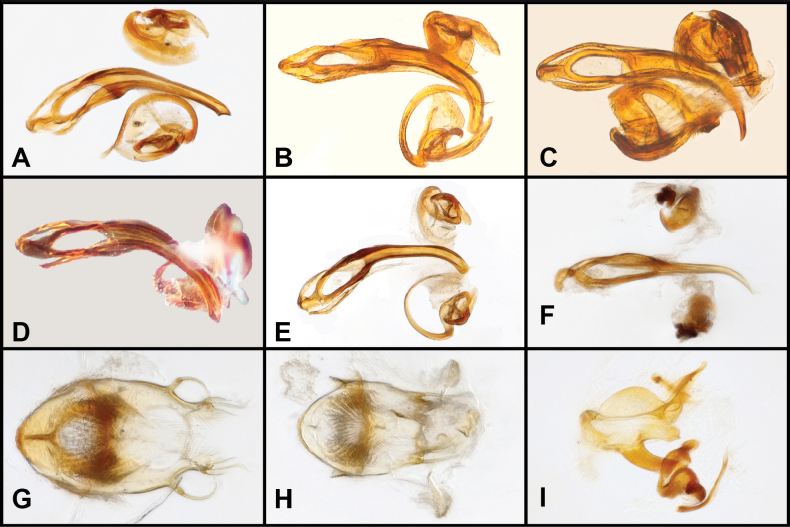
Aedeagus (dorsal view) of *Panabachia* spp. A. *P. papallacta*; B. *P. ananay*; C. *P. cayambi*; D. *P. cryptica*; E. *P. caranqui*; F. *P. patera*; G. *P. vigilans*; H. *P. perdita*; I. *P. ambulans*.

##### Diagnosis.

Head broad, with lateral vertexal foveae deep, non-setose, closer to eyes on each side than to each other; median fovea absent; eyes protuberant but not large, diameter ~2/3 postocular genal width; in ventral view genae projected posteroventrad, rounded (Fig. 5J); two gular foveae present; antennal bases slightly elevated, set off by oblique stria; antennae rather short, antennomere III slightly longer than wide, antennomeres IV–VI beadlike, rounded, VII–IX increasingly transverse, shorter, X short, widening slightly to apex, XI ~2 × as long as X, rounded, with setose depression on inner apex; no antennal dimorphism; male pronotum (Fig. 7A) short, ~1.5 × as wide as long, widest at base, unevenly narrowed to weakly emarginate anterior margin; pronotal disk with large pair of close, deep impressions, a thin carina separating them, with three short, distinct rows of setae at middle and outer edges; outside of major depressions, lateral portion of disk with secondary, shallower impressions just anterad middle, lateral margin slightly angulate near their leading edges; posterior portion of the pronotum slightly swollen, with fine mediobasal fovea and a sparse posterior marginal setal row; two anteroprosternal foveae present; each elytron with four basal foveae, three foveae evenly spaced and one disjunct; discal stria absent; wings present, reduced in size; legs simple; male last sternite convex, with a forked process at the apical margin, setose patches at either side, basally arched setae at middle of apical margin absent; male apical tergite transverse, slightly depressed in the middle. Aedeagus (Fig. 8A) with elongate median lobe and separate accessory sclerites; parameres apparently fused at base and middle of median lobe, briefly separate subbasally; median lobe narrow, with elongate oval basal foramen, narrowed beyond and curved laterad to rounded apex; accessory sclerites large and elaborate, with strongly sclerotized rim surrounding inner hooked disk. TL 1.56–1.59 mm, EW 0.28–0.34 mm.

##### Distribution.

This species is only known from grassland páramo and *Polylepis* forest of the Papallacta region, province of Pichincha, Ecuador.

##### Etymology.

This species is named from the Kichwa word ‘papallacta’, meaning land of potatoes, referring to the highlands where specimens were collected.

##### Remarks.

This species and the following (*P. ananay*) are very closely related, and actually more or less indistinguishable in male genitalia (Fig. 8A vs Fig. 8B). They also share unusual, enlarged posterior genal margins (Fig. 5J, M). However, they are nonetheless clearly separated by genetic data (see Muñoz-Tobar and Caterino 2020) and minor details of pronotal morphology (Fig. 7A vs Fig. 7B). In particular, this species has the lateral angles of the male pronotum further forward and punctate, while the anteromedial cavities are more clearly subdivided into larger posterior and smaller anterior depressions. In *P. ananay*, the main anteromedial cavity is barely divided at all, only by a short rim of setae, with the posterior area smaller than the anterior. It is worth noting that this species is only known from the single locality, whereas *P. ananay* occurs at other localities in Pichincha, Chimborazo, and Carchi provinces.

#### ﻿ Panabachia ananay sp. nov.

Taxon classificationAnimaliaColeopteraStaphylinidae

C1B5DCCF-5364-5BEE-A667-7778CAC6753A

https://zoobank.org/B5F14BE3-1307-4256-8BF0-D9B8B6AA2153

Figs 5M, 7B, 8B

##### Type material.

***Holotype*** • ♂ (QCAZ-I-278830): “ECUADOR: Pichincha, -0.18765, -78.54053, Ruco Pichincha, 3897 m, 22JUN2016, S. Muñoz, A. Romero & S. Myers, Ex. Berlese, páramo” / “Muñoz DNA Voucher, Ex. SIMT296, Morphosp. Pich/Virgen2”; deposited in QCAZI. ***Paratypes*** • 2♀ same data as holotype (SIMT250, SIMT266) (QCAZ-I-278831 to 278832).

##### Other material.

4♀: “ECUADOR: Pichincha, -0.30795, -78.23255, La Virgen, 3694 m, 28JUN2016, S. Muñoz, & R. Tobar, Ex. Berlese, Bosque Polylepis y pajonal” (SIMT274, SIMT276, SIMT277, SIMT247) • 1♀: “ECUADOR: Chimborazo, -1.6400, -78.5071, Releche, 3124 m, 08JUL2016, S. Muñoz & A. Romero, Ex.Berlese (SIMT284) • 1♂: “ECUADOR: Carchi, 0.70587, -77.96642, El Angel, Saladero, 3301 m, 26JUL2016, S. Muñoz & R. Tobar, Ex. Berlese, Páramo de frailejones” (SIMT248) (QCAZ-I-278833 to 278834, 280203 to 280206).

##### Diagnosis.

Head broad, with lateral vertexal foveae deep, non-setose, closer to eyes on each side than to each other; median fovea absent; eyes protuberant but not large, diameter ~2/3 postocular genal width; posteroventral margin of gena projecting, rounded (Fig. 5M); two gular fovea present; antennae rather short, antennomere III slightly longer than wide, IV–VI beadlike, rounded, VII–IX increasingly transverse, shorter, X short, rounded, transverse, XI ~2 × as long as X, rounded, with setose depression on inner apex, no antennal segment differences between male and female; male pronotum (Fig. 7B) slightly wider than long, widest just behind middle due to elevated, angulate processes, with a patch of setae on each posterior edge; posterior edge of disk depressed; median portion of disk with short, deep, transverse depression, lined by setae along lateral and anterior margins, with distinct median fovea within; anterior portion of disk less strongly but more broadly depressed, with setal patches at lateral corners and divergent tufts on either side of anterior edge; deep anterolateral pronotal depressions present at sides, two anteroprosternal foveae present; each elytron with four basal fovea, three fovea evenly spaced and one contiguous fovea, sutural stria complete, discal stria absent; wings present, 5 × the length of elytron; legs simple; male last sternite convex, sparsely setose, with a forked process in the apical margin, three thick basally arched setae at middle of apical margin, male apical tergite transverse, slightly depressed in the middle. Aedeagus (Fig. 8B) with elongate median lobe and separate accessory sclerites; parameres apparently fused at base and middle of median lobe, briefly separate subbasally; median lobe narrow, with elongate oval basal foramen, narrowed beyond and curved laterad to rounded apex; accessory sclerites large and elaborate, with strongly sclerotized rim surrounding inner hooked disk. TL 1.48–1.51 mm, EW 0.36–0.40 mm.

##### Distribution.

This species is known from páramo habitats and *Polylepis* forest in the province of Pichincha, Carchi and Chimborazo, Ecuador.

##### Etymology.

This species’ name comes from a Kichwa term meaning pretty.

##### Remarks.

See preceding remarks to separate this species from the partly sympatric and similar *P. papallacta*.

#### ﻿ Panabachia cayambi sp. nov.

Taxon classificationAnimaliaColeopteraStaphylinidae

93BB3CCC-16F0-57B7-8364-CF56B2A61C88

https://zoobank.org/6345BC19-5755-495B-A48D-84714B412D3B

Figs 5C, 7C, 8C

##### Type material.

***Holotype*** • ♂ (QCAZ-I-280207): “ECUADOR: Pichincha, -0.03502, -78.0601, Cayambe, 3743 m, 01JUL2016, S. Muñoz, Berlese, páramo” / “Muñoz DNA Voucher, Ex. SIMT281, Morphosp. Cayambe3”; deposited in QCAZI. ***Paratypes*** (2♂, 7♀) • 6: same data as holotype • 3: same locality as holotype but collected on 28-Dec-2016 (SIMT254-255, SIMT278 to 280, SIMT282, SIMT309 to 311) (QCAZ-I-280208 to 280216).

##### Diagnosis.

Head broad, with lateral vertexal foveae deep, non-setose, closer to eyes on each side than to each other; two median foveae; eyes protuberant but not large, diameter ~2/3 postocular genal width; two gular foveae present; antennae short, antennomere III slightly longer than wide, IV–VI beadlike, rounded, VII–IX increasingly transverse, shorter, X short, rounded transverse, XI ~3 × as long as X, rounded, with setose depression on inner apex, no antennal segment differences between male and female; male pronotum (Fig. 7C) wider than long, widest toward front, with anterior part of lateral margins rounded, slightly constricted before base, basal margin slightly widened and elevated; small, distinct median subbasal fovea present; anteromedian portion of disk with deep, broad, weakly subdivided, ovoid depression, with dense setal row along posterior margin, sparser setal row at middle of anterior margin, and two small, dense tufts of setae along midline; small, shallow secondary pronotal depressions are present along either side of median depression; two anteroprosternal foveae present; each elytron with four basal foveae, three foveae evenly spaced and one disjunct; sutural stria complete; discal striae absent; wings present, 5 × the length of elytron; legs simple; male last sternite (Fig. 5C) convex, sparsely setose, with a narrow, tongue-like process on the apical margin; male apical tergite transverse, slightly depressed in the middle. Aedeagus (Fig. 8C) with elongate median lobe and separate accessory sclerites; parameres apparently fused to basal apodeme and to middle of median lobe, separate for much of basal ¼ of aedeagal length; median lobe narrow, with elongate, basally rounded distally subquadrate basal foramen; tegmen narrowed beyond and strongly curved laterad to subacute apex; accessory sclerites large and elaborate, each with articulated pair of strongly sclerotized arcs, one (left side) C-shaped, with series of basal serrations. TL 1.46–1.59 mm, EW 0.37–0.40 mm.

##### Distribution.

This species is known from páramo habitats on Mt. Cayambe, province of Pichincha, Ecuador.

##### Etymology.

The name of this species acknowledges the Cayambi people, indigenous to Pichincha, Imbabura, and Napo provinces.

#### ﻿ Panabachia cryptica sp. nov.

Taxon classificationAnimaliaColeopteraStaphylinidae

EC93944E-F2EA-55AC-A71E-AA70440B0D6D

https://zoobank.org/98D1316E-0AD0-4FA5-8EF1-56E45B73FF56

Figs 7D, 8D

##### Type material.

***Holotype*** • ♂ (QCAZ-I-280217): “ECUADOR: Chimborazo, -2.18775, -78.5210, Atillo, 3501 m, 7JUL2016, S. Muñoz & A. Romero, Ex.Berlese” / “Muñoz DNA voucher, SIMT287, Morphosp. Atillo5”; deposited in QCAZI. ***Paratypes*** (1♂, 2♀) • same data as holotype (SIMT 285 to 286) (QCAZ-I-280218 to 280220).

##### Diagnosis.

Head broad, with vertexal foveae deep, non-setose, closer to eyes on each side than to each other; vertexal sulcus present on the posterior portion of the head between vertexal fovea; two gular foveae present; eyes protuberant but not large, diameter ~2/3 postocular genal width; antennae rather short, antennomere III slightly longer than wide, IV–VI beadlike, rounded, VII–IX increasingly transverse, shorter, X short, rounded, transverse, XI ~3 × as long as X, rounded, with setose depression on inner apex, no antennal segment differences between male and female; male pronotum (Fig. 7D) approx. as long as wide, with sides more or less evenly rounded; posterior 1/2 of disk evenly rounded but with deep, small median fovea; anteromedian portion of disk with deep, transversely ovoid depression, with pair of setal tufts at posterolateral corners, and small transverse tuft of setae within; anterior margin of median depression produced posterad forming narrow rim over anterior portion of depression; lateral portion of pronotal disk with deep, round depression slightly posterolaterad median depression, two anteroprosternal foveae present; each elytron with four basal foveae, three foveae evenly spaced and one disjunct; discal stria absent; apical elytral stria incomplete; wings present, 3 × length of elytron; legs simple; male last sternite short, convex, weakly emarginate along apical margin, sparsely setose; male apical tergite transverse, slightly depressed in the middle. Aedeagus (Fig. 8D) with elongate median lobe and separate accessory sclerites; parameres apparently fused to basal apodeme and to middle of median lobe, separate for basal ~1/5 of aedeagal length; median lobe narrow, with elongate ovoid basal foramen; tegmen narrowed beyond and curved laterad to obliquely truncate apex; accessory sclerites large and asymmetrical. TL 1.55–1.56, EW 0.41–0.42.

##### Distribution.

This species is only known from grassland and shrub páramo around the Atillo lakes, province of Chimborazo, Ecuador.

##### Etymology.

This species’ name refers to the partially covered male pronotal excavation.

#### ﻿ Panabachia caranqui sp. nov.

Taxon classificationAnimaliaColeopteraStaphylinidae

AB0E2120-2F18-5053-8B18-3778D4CBC863

https://zoobank.org/CAB8A1D3-14AB-41DB-9B0B-7B31E5053162

Figs 5N, O, 8E

##### Type material.

***Holotype*** • ♂ (QCAZ-I-280221): “ECUADOR: Imbabura, 0.14517, -78.27922, Mojanda, 3715 m, 12JUL2016, S. Muñoz, A. Romero, Ex. Berlese, leaf litter and moss” / “Muñoz DNA voucher, SIMT300, Morphosp. Mojanda6”; deposited in QCAZI.

##### Diagnosis.

Head broad, with lateral vertexal foveae deep, non-setose; median fovea absent; eyes protuberant, small, diameter ~1/3 of the postocular genal length; antennae short, antennomere III small, 1/2 the size of antennomeres I and II, slightly longer than wide, antennomeres IV–VI beadlike, rounded, VII–IX increasingly transverse, shorter, antennomere X short, rounded, transverse, XI ~2 × as long as X; male pronotum simple, without secondary sexual modifications, slightly wider than long, widest toward front; two anteroprosternal foveae present, non-setose; each elytron with four basal foveae, inner three foveae evenly spaced, humeral-most disjunct; wings present, 4 × length of elytra; male last sternite (Fig. 5O) transverse, convex, sparsely setose, with dense row of six flattened setae at middle of apical margin; male apical tergite (Fig. 5N) transverse, slightly depressed in the middle; legs simple. Aedeagus (Fig. 8E) with elongate median lobe and separate accessory sclerites; parameres fused to basal apodeme, narrowing apically but apparently ending free just beyond midpoint of tegmen; median lobe narrow, with very elongate ovoid basal foramen; tegmen narrowed beyond and curved laterad to rounded apex; accessory sclerites slightly asymmetrical, each with long curved inner arch, that of left side thinner and more elongate than that of right; this arch arising from perpendicular complex of hooks, that of right side more robust and with broader base. TL 1.63 mm, EW 0.39 mm.

##### Distribution.

This species is only known from shrub páramo on the slopes of Mt. Mojanda, province of Imbabura, Ecuador.

##### Etymology.

The name of this species acknowledges the Caranqui people, indigenous to Pichincha and Imbabura provinces.

#### ﻿ Panabachia patera sp. nov.

Taxon classificationAnimaliaColeopteraStaphylinidae

0BD96324-30AB-57DF-8EA9-BA8388098054

https://zoobank.org/6C54167B-F474-4E94-8F70-42B5D3E79E46

Figs 3G, 5D, 7E, 8F

##### Type material.

***Holotype*** • ♂ (MECN-EN 40877): “ECUADOR: Pichincha, Quito, Cerro Atacazo, -0.347, -78.611, m, Arbustivo quemado, 01-dic-2018, J. Obregón” / “PTA-196” / “Caterino DNA voucher, Ext. MSC-12820, Morphosp. Panabachia_Atacazo” / “MECN-EN 40877”; deposited in MECN. ***Paratypes*** (4♂, 7♀, all same locality as type) • 3: same data as holotype • 4: 1-Nov-2018 • 1: 1-May-2019 • 2: 1-Feb-2019 • 1: 1-Mar-2019 (MECN-EN 40878 to 40888).

##### Diagnosis.

Head (Fig. 3G) broad, densely setose, with lateral vertexal foveae deep, setose, closer to eyes on each side than to each other, median fovea absent, eyes protuberant but not large, diameter approx. equal to postocular genal width; two gular foveae present; antennal bases elevated, set off by oblique stria; antennae short, antennomere III slightly longer than wide, antennomere IV–VI beadlike, rounded, VII–X increasingly transverse, shorter, antennomere XI short, ~2 × as long as X, rounded, densely setose, with depression on inner apex; no antennal differences between male and female; male pronotum (Fig. 7E) narrow, elongate, subrectangular, basal 2/3 widest, sides slightly rounded, then weakly constricted, with anterior portions of lateral margins widened slightly to front; anterior pronotal margin outwardly arcuate; posterior portion of pronotal disk with broad circular depression, lacking setae within but with small basomedial fovea, setose along lateral margins and with pair of inwardly directed setal tufts at anteromedial edge; anterior portion of pronotal disk with pair of small impressions at middle and pair of slightly larger impressions at sides; two anteroprosternal foveae present; each elytron with four basal foveae, lateral foveae evenly spaced, discal stria absent; wings present; last male sternite (Fig. 5D) convex, male apical tergite transverse, slightly depressed in the middle, legs simple. Aedeagus (Fig. 8F) with elongate median lobe and separate accessory sclerites; parameres not evident; median lobe with narrow, ventrally bent basal apodeme and large elongate oval basal foramen, apical 1/2 narrow, curved to acute apex; accessory sclerites compact, with sclerotized basal knob articulated with elongate, somewhat bean-shaped apical portion, its apex distinctly hooked. TL 1.46–1.57 mm, EW 0.36–0.39 mm.

##### Distribution.

This species is known only from páramo habitats on Cerro Atacazo, just southwest of Quito, in Pichincha province, Ecuador.

##### Etymology.

This species’ name, derived from the Latin, refers to its ‘bowl-shaped’ male pronotal modifications.

##### Remarks.

This species is sympatric with *Panabachia urbana*. The males are easily separated by the fact that this species has strong pronotal dimorphism, being elongate, subquadrate, and deeply foveate (Fig. 7E), while in *P. urbana* the males’ pronota are not modified. Both may be recognized as males by the broad and setose last abdominal tergite and ventrite (Fig. 5D). Females of *P. urbana* have not been identified. But we would expect its females to be broader, especially the pronotum, and lighter.

#### ﻿ Panabachia vigilans sp. nov.

Taxon classificationAnimaliaColeopteraStaphylinidae

8306D0FF-A588-58D8-857D-4407D79EC5D4

https://zoobank.org/8CA34F81-0BB0-4230-83EF-DB9593126B43

Figs 3H, 5F, 7F, 8G

##### Type material.

***Holotype*** • ♂ (ZSFQ-i23412): “ECUADOR: Pichincha 0.0182°N, 78.6372°W, El Pahuma Orchid Res., 28.v-1.vi.2011, 2500 m, Forest litter sifting AT1337” / “Caterino DNA voucher, Ext. MSC-12624, Morphosp. ElP.A.023”; deposited in ZSFQ. ***Paratypes*** (5♂, 3♀) • 5: same data as holotype (ZSFQ-i23413-i23417) • 3 (same general locality as holotype): 7-8-Aug-2008, epiphyte moss sifting (LSAM 0216733, LSAM0216775, LSAM0216778, the last deposited in MECN).

##### Diagnosis.

Head smooth, with small but distinct lateral vertexal foveae, no median fovea; eyes protuberant but not large, diameter ~1/2 length of genae; antennal bases elevated with small oblique stria at the inner base; antennae compact, antennomere III slightly elongate, IV–VI rounded, subquadrate, V slightly wider, VII shorter, rounded, VIII–XI forming loose club, VIII–X transverse, with sharp lateral margins, XI ~3 × as long as X, rounded, with setose depression on inner apex; male pronotum (Fig. 7F) with complex modification on anterior 1/2, elevated at middle, depressed on either side with upper edge of depression distinctly carinate, upper posterior edge of transverse anteromedial impression carinate, with two small tubercles bearing apical tufts of setae; anterior edge of transverse impression produced posterad, vertically carinate medially, densely setose on sides; elytra with fine microsculpture appearing matte; each elytron with sutural and two close dorsobasal foveae, sutural stria complete, discal stria absent; subhumeral foveae and striae absent; male mesotrochanter very weakly dentate; last abdominal ventrite (Fig. 5F) slightly depressed at middle, with short diverging tufts of setae at middle of posterior margin. Aedeagus (Fig. 8G) comprising one piece, broad flattened basal bulb with small subtriangular basal foramen; strongly sclerotized transverse bridge present just anterad basal foramen with laterally projecting digitiform processes bearing long apical setae; apex of median lobe with dorsolateral elevated setal bases projecting obliquely, each bearing a single thick seta, and apicoventral flanges with setose inner and apical margins. TL 1.65 mm, EW 0.34 mm.

##### Distribution.

This species is known only from the El Pahuma Orchid Reserve, northwest of Quito in Pichincha province, Ecuador.

##### Etymology.

The name of this species is a reference to the ‘receiver-dish’-like depressions on the sides of the male pronotum (Fig. 7F), suggesting it is always aware.

#### ﻿ Panabachia perdita sp. nov.

Taxon classificationAnimaliaColeopteraStaphylinidae

6B91A648-C1EB-575A-AA22-162DF0EE0183

https://zoobank.org/6CBC0EF0-C493-4274-AB59-91A15D6B6C88

Figs 3I, 5G, 7G, 8H

##### Type material.

***Holotype*** • ♂ (ZSFQ-i23418): “ECUADOR: Pichincha, 0.0182°N, 78.6372°W, El Pahuma Orchid Res., 28.v-1.vi.2011, FIT, 2200–2400 m. AT1329, M. Caterino, A. Tishechkin” / “Caterino DNA voucher, Ext. MSC-12640, Morphosp. ElP.A.039”; deposited in ZSFQ.

##### Diagnosis.

Head broad, with lateral vertexal foveae deep, non-setose, closer to eyes on each side than to each other; antennal bases slightly swollen but not set off by striae; antennae rather short, antennomere III slightly longer than wide, IV–VII short and beadlike, VIII slightly transverse, IX–XI forming a loose club, IX transverse, X (in male) with flattened densely setose upper surface, terminal antennomere bluntly acuminate, with small setose depression on anterior surface; pronotum subangulate laterally, widest just basad of middle, lateral and median basal foveae present; male pronotal disk (Fig. 7G) with simple small, round depression in anterior 1/2, glabrous within, with just a few posteriorly directed setae along anterior margin; each elytron with three dorsobasal foveae, one at base of sutural stria, other as a closely set pair near humerus; most male ventrites (Fig. 5G) flattened at middle, first visible sternite with dense patch of setae along posterior margin, slightly clustered to sides, posterior marginal fringes of setae on 2^nd^ and 3^rd^ visible ventrites thinner but also evident; last visible ventrite short, shallowly depressed, with small median cluster of convergent setae; last abdominal tergite strongly curved ventrad, surface matte, slightly emarginate apically. Aedeagus (Fig. 8H) broad and somewhat inflated at base, with strongly sclerotized ventrobasal bridge, the bridge with rounded, separate setose lateral lobes and triangular posterolateral lobes, connected by membrane to separate, irregular setose sclerites; median lobe broadly emarginate apically, dorsally on each side with elevated setal base, and very thick apically directed seta; venter of median lobe with long, laterally and apically fimbriate median plate; apical ventral margin with bilateral lobes bearing tufts of long setae as well as expanded, membraneous microdentate flaps. TL 1.48 mm, EW 0.38 mm.

##### Distribution.

This species is known only from the El Pahuma Orchid Reserve, northwest of Quito in Pichincha province, Ecuador.

##### Etymology.

The name of this species mean ‘lost’ or ‘outcast’ due to the fact that it seems to be a phylogenetic outlier (and maybe interloper) in this genus.

#### ﻿ Panabachia ambulans sp. nov.

Taxon classificationAnimaliaColeopteraStaphylinidae

D9632324-995E-52A9-8C97-77A6CB816079

https://zoobank.org/02D95330-F82F-4A20-AE7B-40FEAB8454E8

**Figure 9.**
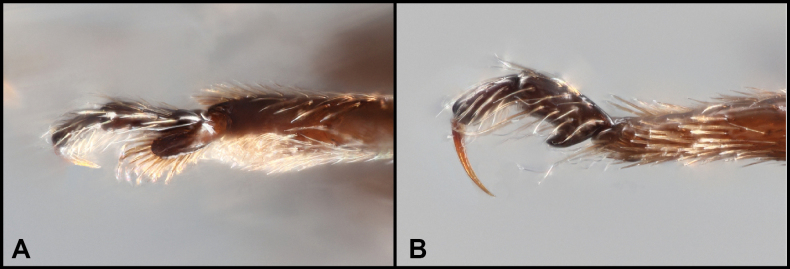
A. protarsus and B. mesotarsus of *P. ambulans*.

Figs 3J, 5H, 7H, 8I, 9

##### Type material.

***Holotype*** • ♂ (QCAZ-I-280222/MECN-EN-40689): “ECUADOR: Chimborazo, Páramo de Atillo, 3501 m, 02°11.265'S, 78°31.2601'W, 08-VII-2016, Berlese, S. Muñoz & A. Romero” / “Caterino DNA voucher, Ext. MSC-13035, Morphosp. Atillo”; deposited in QCAZI.

##### Diagnosis.

Body (Fig. 3J) large, bronzy brown, with fine, mostly recumbent setae; head broadly subquadrate posteriorly, slightly narrowed to front; vertexal foveae distinct but not too deep; antennal bases slightly swollen in front of short, oblique striae; eyes rather small, diameter less than post-ocular genal length; antennae long, scape ~2 × as long as wide, tapering slightly, antennomere II cylindrical, III conical, longer than II; IV–VII all elongate-cylindrical, somewhat varied in length, VIII shorter, IX–XI gradually enlarged, forming loose club; pronotum slightly longer than wide, widest near front, narrowed abruptly to a narrow anterior collar, more gradually to base; pronotal disk (Fig. 7H) with distinct median and very faint lateral foveae, setiferous punctures slightly denser toward front; male pronotum unmodified; elytra short, sides evenly tapered to humeri (wingless), each with four basal foveae, the lateral pair approximate and sharing a depression; 1^st^ male protarsomere (Fig. 9A) enlarged into distinct setose lobe beneath and along lateral margin of 2^nd^; male 2^nd^ mesotarsomere (Fig. 9B) enlarged, with distinct, blunt ventral tooth, 3^rd^ mesotarsomere thin, short, mesotarsal claw single, very elongate, able to chelate with 2^nd^ tarsomere’s ventral tooth; all tarsal claws unpaired, long; abdomen large; male last ventrite (Fig. 5H) broadly and deeply depressed, densely setose, opposing surface of last tergite similarly depressed and setose. Aedeagus (Fig. 8I) radically asymmetrical, basal foramen oval, parameres indistinct; tegmen with three irregularly trilobed, left lobe long, curved, articulating apically with enlarged, weakly coiled accessory sclerite, this sclerite with a separate, thin distal process; median lobe of tegmen shortest, ending bluntly, free, between enlarged lateral lobes; right lobe of tegmen arising from a large, arcuate basal lamina, tip flattened, curving ventrolaterad, with acute, dorsal subapical spine, tip recurved dorsad, bluntly rounded; second accessory sclerite free near apex of right tegmental lobe, simple, elongate, curved. TL 1.88 mm, EW 0.38 mm.

##### Distribution.

This species is only known from grassland and shrub páramo around the Atillo lakes, province of Chimborazo, Ecuador.

##### Etymology.

The name of this species means ‘walker’, a reference to its flightlessness.

##### Remarks.

This species is unique in numerous respects. It is considerably larger than any other, and the male’s flightlessness (and clearly associated body proportion changes - the bulky abdomen in particular; Fig. 3J) see no parallels in other species. The lack of male pronotal modifications recurs in a number of other species, none of which appear to be closely related to this species or to each other. The male shows tarsal modifications seen in no other species. While a few other males exhibit slightly modified setae on the basal protarsomere(s) (e.g. *P. salebrosa*), none have the basal tarsomere itself modified, and the enlarged tarsal claws that can chelate with the 2^nd^ mesotarsomere are completely unique. Finally, the aedeagus is very difficult to homologize with any other. Yet, upon closer inspection, it exhibits a basally flattened tegmen, with small oval basal foramen, and it has free accessory sclerites. The tegmen is highly asymmetrical, and subdivided into a trio of lateral lobes, one of which has become closely associated with a highly modified accessory sclerite. The second accessory sclerite appears to have been reduced to a small vestige.

## ﻿Discussion

This study has revealed a surprising diversity of *Panabachia* species from a remarkably small area, considering that the genus is distributed across montane regions from Costa Rica south to Bolivia. Undoubtedly large numbers of species remain to be discovered as the high Andes and Central American cordillera are better documented through litter sifting techniques.

Most of the species documented here are known from single localities, despite fairly thorough surveys by the senior author and others working in the region (e.g. Moret 2005, 2009; Moret et al. 2016) This is not a surprising result, as mountain ecosystems in the northern Andes are characterized by high numbers of endemic species, partially a result of complex geological and paleoclimatic events during the Andes formation (Antonelli et al. 2018; Pérez-Escobar et al. 2022; Romoleroux et al. 2023), as well as influences from adjacent hyperdiverse lower-elevation regions such as Amazonia (Antonelli et al. 2018; de Meyer et al. 2022) and Choco areas (Pérez-Escobar et al. 2019). High rates of diversification have been reported for both páramo (Madriñán et al. 2013; Cortés et al. 2018) and montane forest lineages (Hutter et al. 2017; Karger et al. 2021; Vieu et al. 2022), where species are usually restricted to narrow and fragmented elevational bands. Unfortunately, these diverse upland faunas are increasingly threatened by climate change and changes of land use (Karger et al. 2021; Romoleroux et al. 2023).

The main clades highlighted in Muñoz-Tobar and Caterino (2020) correspond well to major morphological groupings based on genitalia. The ‘upper’ (in their fig. 4) clade, corresponding to their numbered species 1–6, includes all those species with a long, curving median lobe, while the lower clade (their species 7–17) corresponds to those species with a shorter, broader, flat median lobe. While we have not had the opportunity to assemble a more complete molecular data set from the larger set of now-known species, we can predict that the as-yet unsequenced species *P. pahuma*, *P. pastazae*, *P. urbana*, *P. carltoni*, and *P. falini* will fall out among the latter, while *P. patera* will be resolved in the former. Determining placements for the morphological outliers, *P. perdita*, *P. vigilans*, and *P. ambulans* is harder to predict, although the very dissimilar aedeagal morphology of the first two would suggest stem or sister group placements. Those species with a longer, curved aedeagus are known only from páramo, while flatter aedeagi are known in both cloud forest and páramo species. We have now also seen new species from Colombia and Venezuela, not described here, that have a flattened aedeagus with separate accessory sclerites, so presumably are members of the ‘flattened aedeagus’ clade. So far the type with an elongated and curved tegmen are limited to the Ecuadorean highlands. Whether this clade extends to higher regions of more distant Andean regions remains to be seen.

It is interesting to observe that species lacking male pronotal modifications are found in both major clades. Without even having a hypothetical function for these modifications, it is difficult to speculate on reasons for convergent losses, although if mate recognition were involved, it might be interesting to look more carefully at variation in these characters in communities where more than one *Panabachia* species was known, such as La Virgen, Atillo, and Mojanda. There are also similar modifications in both clades, with transverse creases, secondary lateral depressions, and median setose fringes, suggesting common modes of employment across considerable phylogenetic space. We leave such questions for future students.

Studies of alpine beetle faunas in the Andes are in their preliminary stages. But our findings, showing high diversity in a novel beetle lineage from páramo and montane forest in Ecuador, echo those findings available to date. Previous studies have also indicated that tropical alpine ecosystems in the Andes hold a high number of endemic species (Pérez-Escobar et al. 2022; Romoleroux et al. 2023), mostly undescribed among invertebrate groups. Some of the better-studied examples include the ground beetles, for which we have a good understanding of diversity and distribution in Ecuadorian páramo, with more than 224 páramo specialists (Moret 2009; Atiencia-Puca et al. 2023). In a few other groups, such as broad nosed weevils, páramo specialists have been described in the genus *Obrieniolus* del Río (Coleoptera, Curculionidae, Entiminae) from Peru (del Río and Lanteri 2011), as well as new species in the genus *Leschenius* del Rio (del Río and Marvaldi 2022) from Ecuadorian páramo. Among the diving beetles, one new species and seven subspecies in the genus *Liodessus* (Coleoptera, Dytiscidae) from eastern Colombian páramo have been identified (Balke et al. 2023). Similar patterns of high diversity have been described for hover flies from Colombian páramo complexes, with six genera and 37 species endemic to the area of study (Montoya et al. 2021). These studies focused on montane Andean biodiversity clearly indicate a high richness, and show the importance of conserving high elevation ecosystems. Most work to date (including this one) has been limited to particular countries. Integrative regional studies that encompass the range of Andean alpine habitats should be a high priority to reveal broader patterns of diversity and evolution.

## Supplementary Material

XML Treatment for Panabachia

XML Treatment for Panabachia pahuma

XML Treatment for Panabachia trifecta

XML Treatment for Panabachia inornata

XML Treatment for Panabachia amica

XML Treatment for Panabachia winku

XML Treatment for Panabachia ayauma

XML Treatment for Panabachia pastazae

XML Treatment for Panabachia romeroi

XML Treatment for Panabachia uktu

XML Treatment for Panabachia salebrosa

XML Treatment for Panabachia urbana

XML Treatment for Panabachia carltoni

XML Treatment for Panabachia falini

XML Treatment for Panabachia papallacta

XML Treatment for Panabachia ananay

XML Treatment for Panabachia cayambi

XML Treatment for Panabachia cryptica

XML Treatment for Panabachia caranqui

XML Treatment for Panabachia patera

XML Treatment for Panabachia vigilans

XML Treatment for Panabachia perdita

XML Treatment for Panabachia ambulans

## ﻿Additional information

### Use of AI

No use of AI was reported.

### Author ORCIDs

Sofia I. Muñoz-Tobar https://orcid.org/0000-0002-4609-3703

Michael S. Caterino https://orcid.org/0000-0002-2597-5707

### Data availability

All of the data that support the findings of this study are available in the main text.

## References

[B1] Antonelli A Kissling WD Flantua SG Bermúdez MA Mulch A Muellner-Riehl AN Kreft H Linder HP Badgley C Fjeldså J Fritz SA Rahbek C Herman F Hooghiemstra H Hoorn C (2018) Geological and climatic influences on mountain biodiversity. Nature Geoscience 11(10): 718–725. 10.1038/s41561-018-0236-z

[B2] Asenjo A Klimaszewski J Chandler DS Fierros-López HE Vieira JS (2019) Staphylinidae (Insecta: Coleoptera) in Latin America: synopsis, annotated catalog, diversity and distribution. Zootaxa 4621(1): 1–406. 10.11646/zootaxa.4621.1.1 31716285

[B3] Atiencia-Puca E Barragán Á Guevara D (2023) Revisión bibliográfica de Carabidae (Coleoptera) en los Andes del Ecuador. Revista Ecuatoriana de Medicina y Ciencias Biológicas 44(2): 11–31. 10.26807/remcb.v44i2.969

[B4] Balke M Neven K Villastrigo A Ospina-Torres R Prieto C Gutierrez Rubiano N Lotta I Dueñas LF Hendrich L (2023) Eastern Colombian Páramo *Liodessus* Guignot, 1939 diving beetles are genetically structured, but show signs of hybridization, with description of new species and subspecies (Coleoptera, Dytiscidae). ZooKeys 1143: 165–187. 10.3897/zookeys.1143.97461 37234279 PMC10207932

[B5] Carlton C Dean M Tishechkin A (2004) Diversity of two beetle taxa at a western Amazonian locality (Coleoptera: Histeridae; Staphylinidae, Pselaphinae). Coleopterists Bulletin 58(2): 163–170. 10.1649/603

[B6] Chandler DS (1992) Catalogue of the short-winged mold beetles from Panama (Coleoptera: Pselaphidae). In: Quintero D Aiello A (Eds) Insects of Panama and Mesoamerica selected studies.Oxford University Press, NY, 339–344. 10.1093/oso/9780198540182.003.0022

[B7] Cortés AJ Garzón LN Valencia JB Madriñán S (2018) On the causes of rapid diversification in the Páramos: Isolation by ecology and genomic divergence in *Espeletia*. Frontiers in Plant Science 9: 1700. 10.3389/fpls.2018.01700 PMC6294130 30581444

[B8] de Meyer AP Ortega-Andrade HM Moulatlet GM (2022) Assessing the conservation of eastern Ecuadorian cloud forests in climate change scenarios. Perspectives in Ecology and Conservation 20(2): 159–167. 10.1016/j.pecon.2022.01.001

[B9] del Río M Lanteri A (2011) *Obrieniolus*, a new monotypic genus of Naupactini (Coleoptera, Curculionidae, Entiminae) from the Peruvian Andes and its phylogenetic placement. ZooKeys 102: 51–60. 10.3897/zookeys.102.1240 PMC3131080 21747674

[B10] del Río MG Marvaldi AE (2022) On the Andean genus *Leschenius* (Coleoptera: Curculionidae: Entiminae): Updated phylogeny, with a new species from Ecuador, discovery of males, and larval description of the potato weevil *Leschenius vulcanorum*. PeerJ 10: e12913. 10.7717/peerj.12913 PMC8840050 35186491

[B11] del Río MG Marvaldi AE Lanteri A (2012) Systematics and cladistics of a new Naupactini genus (Coleoptera: Curculionidae: Entiminae) from the Andes of Colombia and Ecuador. Zoological Journal of the Linnean Society 166(1): 54–71. 10.1111/j.1096-3642.2012.00833.x

[B12] Hutter CR Lambert SM Wiens JJ (2017) Rapid diversification and time explain amphibian richness at different scales in the Tropical Andes, Earth’s most biodiverse hotspot. The American Naturalist 190(6): 828–843. 10.1086/694319 29166157

[B13] Karger DN Kessler M Lehnert M Jetz W (2021) Limited protection and ongoing loss of tropical cloud forest biodiversity and ecosystems worldwide. Nature Ecology & Evolution 5(6): 854–862. 10.1038/s41559-021-01450-y 33927369

[B14] Madriñán S Cortés AJ Richardson JE (2013) Páramo is the world’s fastest evolving and coolest biodiversity hotspot. Frontiers in Genetics 4: 192. 10.3389/fgene.2013.00192 PMC3793228 24130570

[B15] Montoya AL Parra JL Wolff M (2021) Structure and diversity of hoverflies (Diptera: Syrphidae) in northwestern Colombian Paramos: towards the identification of bioindicator species in the Tropical Andes. Journal of Insect Conservation 25(5): 809–828. 10.1007/s10841-021-00346-3

[B16] Moret P (2005) Los Coleópteros Carabidae Del Páramo En Los Andes Del Ecuador; Museo de Zoología; Centro de Biodiversidad y Ambiente; Escuela de Biología; Pontificia Universidad Católica del Ecuador: Quito, Ecuador, 11–273.

[B17] Moret P (2009) Altitudinal distribution, diversity and endemicity of Carabidae (Coleoptera) in the páramos of Ecuadorian Andes. Annales de la Société Entomologique de France 45(4): 500–510. 10.1080/00379271.2009.10697632

[B18] Moret P Aráuz MDLÁ Gobbi M Barragán Á (2016) Climate warming effects in the tropical Andes: First evidence for upslope shifts of Carabidae (Coleoptera) in Ecuador. Insect Conservation and Diversity 9(4): 342–350. 10.1111/icad.12173

[B19] Muñoz-Tobar SI (2019) Weak genetic differentiation among populations of the Andean ground beetle *Pelmatellus columbianus* (Reiche, 1843) (Coleoptera: Carabidae). Coleopterists Bulletin 73(2): 411–427. 10.1649/0010-065X-73.2.411

[B20] Muñoz-Tobar SI Caterino MS (2019) The role of dispersal for shaping phylogeographical structure of flightless beetles from the Andes. PeerJ 7: e7226. 10.7717/peerj.7226 PMC6611450 31304068

[B21] Muñoz-Tobar SI Caterino MS (2020) Mountains as islands: Species delimitation and evolutionary history of the ant-loving beetle genus *Panabachia* (Coleoptera, Staphylinidae) from the Northern Andes. Insects 11(64): 1–19. 10.3390/insects11010064 PMC7023032 31968550

[B22] Navarrete-Heredia JL Newton AF Thayer MK Ashe JS Chandler DS (2002) Guía ilustrada para los géneros de Staphylinidae (Coleoptera) de México. Universidad de Guadalajara y CONABIO, México, [xii +] 401 pp.

[B23] Newton AF Gutiérrez-Chacón C Chandler DS (2005) Checklist of the Staphylinidae (Coleoptera) of Colombia. Biota Colombiana 6(1): 1–72. 10.21068/bc.v6i1.148

[B24] Park O (1942) A study in neotropical Pselaphidae. Northwestern University Studies in the Biological Sciences and Medicine, Number 1, 1, Northwestern University, Evanston and Chicago, [x +] 403 pp [21 pls].

[B25] Park O (1945) Further studies in Pselaphidae (Coleoptera) of Mexico and Guatemala. Bulletin of the Chicago Academy of Sciences. 7(7): 331–443.

[B26] Pérez-Escobar OA Lucas E Jaramillo C Monro A Morris SK Bogarín D Antonelli A (2019) The origin and diversification of the hyperdiverse flora in the Chocó biogeographic region. Frontiers in Plant Science 10: 1328. 10.3389/fpls.2019.01328 PMC6910151 31867022

[B27] Pérez-Escobar OA Zizka A Bermúdez MA Meseguer AS Condamine FL Hoorn C Chomicki G (2022) The Andes through time: Evolution and distribution of Andean floras. Trends in Plant Science 27(4): 364–378. 10.1016/j.tplants.2021.09.010 35000859

[B28] Romoleroux K Muriel P Sklenář P Ulloa-Ulloa C Espinel D Romoleroux C (2023) La flora de los Páramos ecuatorianos: orígenes, diversidad y endemismo. In: Hofstede R Mena-Vásconez P Suárez E (Eds) Los Páramos del Ecuador: Pasado, presente y futuro.USFQ PRESS, 218–245. 10.18272/usfqpress.71.c260

[B29] Schneider CA Rasband WS Eliceiri KW (2012) NIH Image to ImageJ: 25 years of image analysis. Nature Methods 9(7): 671–675. 10.1038/nmeth.2089 22930834 PMC5554542

[B30] Sharp DS (1887) Biologia Centrali-Americana, Insecta, Coleoptera, Pselaphidae, Scydmaenidae. Taylor & Francis, London, UK 1–71 [pls 1, 2].

[B31] Vieu JC Hughes CE Kissling J Grant JR (2022) Evolutionary diversification in the hyper-diverse montane forests of the tropical Andes: Radiation of *Macrocarpaea* (Gentianaceae) and the possible role of range expansion. Botanical Journal of the Linnean Society 199(1): 53–75. 10.1093/botlinnean/boab065

